# Sex- and age-specific multimorbidity networks in middle-aged inpatients: a network-based comparative study between China and the United Kingdom

**DOI:** 10.1093/geroni/igaf090

**Published:** 2025-08-18

**Authors:** Yining Bao, Hanting Liu, Qianhui Lu, Yang Sun, Lin Wang, Shu Su, Pengyi Lu, Mengjie Wang, Ting Ma, Xinxin Xie, Wenhua Wang, Liqin Wang, Yuhang Zhai, Fang Lu, Yudong Wei, Rui Li, Miao Ding, Yiqi Yan, Shiwei Jia, Xueli Zhang, Jiangcun Yang, Lei Zhang

**Affiliations:** China-Australia Joint Research Center for Infectious Diseases, School of Public Health, Xi’an Jiaotong University Health Science Center, Xi’an, China; School of Translational Medicine, Faculty of Medicine, Nursing and Health Sciences, Monash University, Melbourne, Australia; Department of Medical Statistics, School of Public Health, Sun Yat-sen University, Guangzhou, China; Department of Epidemiology and Biostatistics, College of Public Health, Zhengzhou University, Zhengzhou, China; Department of Transfusion Medicine, Shaanxi Provincial People’s Hospital, Xi’an, China; Department of Ophthalmology, Eye & ENT Hospital of Fudan University, Shanghai, China; Key laboratory of Myopia and Related Eye Diseases, Chinese Academy of Medical Sciences, Shanghai, China; Clinical Research Management Office, The Second Affiliated Hospital of Chongqing Medical University, Chongqing, China; China-Australia Joint Research Center for Infectious Diseases, School of Public Health, Xi’an Jiaotong University Health Science Center, Xi’an, China; China-Australia Joint Research Center for Infectious Diseases, School of Public Health, Xi’an Jiaotong University Health Science Center, Xi’an, China; Department of Transfusion Medicine, Shaanxi Provincial People’s Hospital, Xi’an, China; Department of Transfusion Medicine, Shaanxi Provincial People’s Hospital, Xi’an, China; Department of Transfusion Medicine, Shaanxi Provincial People’s Hospital, Xi’an, China; Department of Transfusion Medicine, Shaanxi Provincial People’s Hospital, Xi’an, China; Gies College of Business, University of Illinois Urbana-Champaign, Champaign, Illinois, United States; China-Australia Joint Research Center for Infectious Diseases, School of Public Health, Xi’an Jiaotong University Health Science Center, Xi’an, China; China-Australia Joint Research Center for Infectious Diseases, School of Public Health, Xi’an Jiaotong University Health Science Center, Xi’an, China; China-Australia Joint Research Center for Infectious Diseases, School of Public Health, Xi’an Jiaotong University Health Science Center, Xi’an, China; China-Australia Joint Research Center for Infectious Diseases, School of Public Health, Xi’an Jiaotong University Health Science Center, Xi’an, China; Medical College of Yan’an University, Yan’an University, Yan’an, China; Information Department, Shaanxi Provincial People’s Hospital, Xi’an, China; Medical Research Institute, Guangdong Provincial People’s Hospital (Guangdong Academy of Medical Sciences), Southern Medical University, Guangzhou, China; Guangdong Eye Institute, Department of Ophthalmology, Guangdong Provincial People’s Hospital (Guangdong Academy of Medical Sciences), Southern Medical University, Guangzhou, China; Department of Transfusion Medicine, Shaanxi Provincial People’s Hospital, Xi’an, China; School of Translational Medicine, Faculty of Medicine, Nursing and Health Sciences, Monash University, Melbourne, Australia; Phase I Clinical Trial Research Ward, The Second Affiliated Hospital of Xi’an Jiaotong University, Xi’an, China

**Keywords:** Comorbidity pattern, Network analysis, Sex-specific medicine, Hospitalized patients

## Abstract

**Background and Objectives:**

Multimorbidity is increasingly prevalent among the middle-aged population, yet it is largely often overlooked. We aimed to explore and compare the differences in multimorbidity patterns by sex and age among middle-aged inpatients from China and the United Kingdom.

**Research Design and Methods:**

We analyzed 184 133 hospitalization records from Shaanxi, China, and 180 497 from the UK Biobank for ­middle-aged populations. Using network analysis, we examined multimorbidity patterns by sex, age groups (40-44, 45-49, 50-54, and 55-59 years), and countries. We also identified hub diseases in both sex-specific and sex–age-specific networks and their corresponding roles in forming multimorbidity patterns.

**Results:**

In both China and the United Kingdom, males exhibited higher multimorbidity prevalence (China: 58.51% vs 55.33%, 1.06×; United Kingdom: 31.15% vs 29.79%, 1.05×) and greater complexity of multimorbidity patterns (China: 1179 patterns vs 990 patterns, 1.19×; United Kingdom: 438 patterns vs 377 patterns, 1.16×) than females. In sex-specific networks, males in both countries demonstrated the specificity of circulatory, genitourinary, and endocrine/nutritional/metabolic-associated multimorbidity patterns, while females demonstrated specific genitourinary and neoplasm-associated multimorbidity patterns. Hub diseases in these networks are distributed in similar disease categories. In sex–age-specific networks, dominant multimorbidity patterns and hub diseases shifted by age. In males, both countries showed stable but dominating circulatory, endocrine/nutritional/metabolic and digestive-associated multimorbidity patterns with aging. In comparison, Chinese females demonstrated an increase in nervous system-associated multimorbidity patterns and a decrease in genitourinary-associated multimorbidity patterns with ageing; British females demonstrated an increase in mental/behavioral-associated multimorbidity patterns and a stable but dominating ­genitourinary-associated multimorbidity patterns.

**Discussion and Implications:**

In both China and the United Kingdom, males demonstrated more complex multimorbidity than females. With ageing, multimorbidity patterns are stable in males, while females in China and the United Kingdom each develop different and specific multimorbidity patterns. These findings may inform targeted interventions for middle-aged inpatients with multimorbidity by sex and age.

Translational Significance We examine multimorbidity patterns in middle-aged inpatients in China and the United Kingdom through network analysis of over 180 000 hospitalization records in each country. We focus on midlife multimorbidity, as understanding its pattern and early intervention may substantially slow disease progression and improve population health outcomes in later life stages. The study highlights the role of sex and age in affecting these patterns, aiding the development of personalized health strategies for multimorbidity prevention. In addition, the identification of hub diseases provides an important evidence base for targeted prevention of multimorbidity, thereby reducing complications, disease burden, and economic burden on healthcare systems.

## Introduction

Multimorbidity, the coexistence of multiple diseases in an individual,^1^ poses a significant challenge to global health.^2^ Our study aligns with the review recommendations by including both acute and chronic diseases to better understand their interrelationships and potential disease progression process.^3^ The prevalence of multimorbidity is rising globally, increasing from 54% in 2015 to 68% by 2035 in high-income countries like the United Kingdom,^4^ but also in low- and middle-income countries like India and China.^5^ Multimorbidity causes premature death, reduced functionality, lower quality of life, and increased healthcare utilization.^6^ The World Health Organization has identified multimorbidity as a critical area for research and policy,^7^ emphasizing the need to understand its effects across different sexes, ages, and regions.^8–11^

Previous research on multimorbidity across regions, such as Canada versus Australia^10^ and China versus India,^11^ is limited. Small and imbalanced sample sizes (5000-31 000 individuals), and a narrow disease spectrum (a dozen diseases), combined with reliance on self-reported diagnoses that reduce accuracy, highlight significant gaps in understanding national variations. Additionally, previous studies have primarily compared multimorbidity between low- and middle-income countries and between low- and high-income countries,^12^^,^^13^ with limited evidence on multimorbidity in high- and middle-income countries. Our study uses hospital data with International Statistical Classification of Diseases and Associated Health Problems 10th (ICD-10) coding to compare multimorbidity between the United Kingdom (a high-income country) and China (a ­middle-income country), enhancing diagnostic accuracy and encompassing all diseases in ICD-10 Chapters 1-14 to map multimorbidity patterns and construct a comprehensive “panoramic view” of multimorbidity research. Further, China’s public healthcare system prioritizes high-quality resources in tertiary hospitals, while primary care remains underdeveloped.^14^ In contrast, the United Kingdom’s National Health Service (NHS) ensures care coordination through a tiered system.^15^ Understanding multimorbidity across regions helps identify differences in multimorbidity disease profiles between 2 countries, provides evidence to support primary care, reveals economic, cultural, and lifestyle impacts, and guides medical resource allocation.^16–18^

Research indicates that multimorbidity impacts all ages, notably spiking in the middle-aged, but this group is often overlooked in studies.^19^^,^^20^ Further examination of multimorbidity in middle-aged populations is essential because interventions in the early stages of life can reduce the subsequent disease burden of multimorbidity in later life stages. Our study analyzed hospitalization records from Shaanxi, China, for individuals aged 18-59, and from the UK Biobank for middle-aged to elderly adults, focusing our comparative analysis on the overlapping middle-aged group, which is the majority of both populations. Moreover, studying multimorbidity patterns evolution across age groups enhances understanding of multimorbidity progression and highlights multimorbidity patterns at different life stages.^3^^,^^18^^,^^19^ This understanding enables the development of targeted prevention strategies to reduce multimorbidity in specific age groups, allowing healthcare professionals to tailor treatments based on age-specific needs.

Research indicates that multimorbidity prevalence and patterns vary between sexes, with males and females experiencing different diseases due to genetic, hormonal, physiological, behavioral, and sociocultural differences.^21^^,^^22^ Some studies have shown that multimorbidity is more prevalent in females (39.4%) than males (32.8%),^23^ while some studies showed the opposite direction.^19^^,^^20^ Cardiovascular and cancer disease comorbidities were most common in males,^24^ whereas females had a wider range of comorbid mental and physical diseases along with mental health disorders.^19^ Investigating gender differences in multimorbidity patterns promotes personalized treatments and gender-sensitive strategies.^25^ Comprehensive studies comparing multimorbidity across genders by region are scarce.

Our previous study compared multimorbidity networks across different regions for overall populations, males, and females.^26^ Building on the previous study, we further investigate how multimorbidity patterns differ between males and females by analyzing multimorbidity patterns that were shared between the sexes and patterns specific to each of the sexes. In addition, we investigated the changes in multimorbidity patterns by age in both sexes and compared them between the countries. Our results may guide targeted prevention strategies across demographic groups to reduce the disease burden of multimorbidity.

## Methods

### Study participants

Our study analyzed ICD-10 coded disease diagnosis data^27^ from 678 666 participants in Shaanxi, China (1998-2018), with 1 058 398 hospitalization records from the Centralized Hospital Medical Records system,^28^ and 502 414 UK Biobank participants (2006-2010) with 2 372 119 hospitalization records from Hospital Episode Statistics.^29^ Baselines were set at each participant’s first record, with ICD-10 codes sorted into 22 chapters (Supplementary Text S1). Details and inclusion criteria for the datasets are in external sources^28^^,^^30^ (Figures S1 and S2 [see online supplementary material for a color version of this figure], Supplementary Text S2 and S3).

### Inclusion and exclusion criteria

We used consistent inclusion criteria for both datasets, requiring (1) baseline records of each inpatient; (2) health records with ICD-10 codes; (3) ages 40-59 years (middle-aged). Exclusion criteria included records with ICD-10 codes only from Chapters 15 to 22. Only records with ICD-10 codes from Chapters 1 to 14 were retained, which was consistent with previous studies^31^ (Figures S1 and S2 [see online supplementary material for a color version of this figure], Supplementary Text S2 and S3).

### Study population and subpopulations

Our criteria yielded 184 133 hospitalization records from China and 180 497 from the United Kingdom. Gender analysis showed 103 334 Chinese and 79 652 UK male inpatients, and 80 799 Chinese and 100 845 UK female inpatients. Each dataset was divided into 8 subpopulations by sex and 4 age categories (40-44, 45-49, 50-54, 55-59) (Table 1).

**Table 1.** The number of health records, disease and multimorbidity conditions, and characteristics of complete multimorbidity network and hub diseases’ associated network in the Chinese and UK inpatients by sex and age.

Variable	Whole inpatients	Male inpatients					Female inpatients				
	Whole	Total (40-59 years)	40-44 years	45-49 years	50-54 years	55-59 years	Total (40-59 years)	40-44 years	45-49 years	50-54 years	55-59 years
**China**											
**Number of health records**	184 133	103 334	30 167	31 655	25 402	16 110	80 799	20 956	24 751	21 550	13 542
**Numbers of diseases and diagnoses**	341, 440 702	320, 237 746	279, 39 536	291, 69 212	298, 61 225	293, 43 010	297, 169 760	238, 35 632	277, 49 411	266, 47 299	256, 31 359
**Number of inpatients with a single disease (% of the total number of inpatients)**	78 963 (42.88%)	42 871 (41.49%)	14 090 (46.71%)	13 566 (42.86%)	25 402 (38.27%)	5494 (34.10%)	36 092 (44.67%)	10 620 (50.68%)	11 182 (45.18%)	9034 (41.92%)	5256 (38.81%)
**Number of inpatients with multimorbidity ≥2 conditions) (% of the total number of inpatients)**	105 170 (57.12%)	60 463 (58.51%)	16 077 (53.29%)	18 089 (57.14%)	15 681 (61.73%)	10 616 (65.90%)	44 707 (55.33%)	10 336 (49.32%)	13 569 (54.82%)	12 516 (58.08%)	8286 (61.19%)
**Number of nodes and edges in the complete multimorbidity network**	341, 1367	320, 1179	279, 735	291, 808	298, 773	293, 695	297, 990	238, 492	277, 646	266, 640	256, 552
**Number of nodes and edges in the hub diseases network**	10, 32	10, 35	10, 25	10, 31	10, 27	10, 26	10, 31	10, 18	10, 19	10, 18	10, 20
**Number of nodes and edges in hub diseases’ associated network (% of the complete multimorbidity network)**	193 (56.60%), 483 (35.33%)	173 (54.06%), 410 (34.78%)	121 (43.37%), 238 (32.38%)	119 (40.89%), 248 (30.69%)	118 (39.60%), 218 (28.20%)	103 (35.15%), 194 (27.91%)	160 (53.87%), 347 (35.05%)	97 (40.76%), 168 (34.15%)	109 (39.35%), 202 (31.27%)	99 (37.22%), 193 (30.16%)	83 (32.42%), 160 (28.99%)
**Frequency of multimorbidity patterns in the complete multimorbidity network**	287 195	174 985	31 588	42 716	43 273	32 102	99 086	14 091	23 931	24 408	19 272
**Frequency of multimorbidity patterns in the hub diseases network**	55 044	41 501	5684	11 130	10 611	6416	19 047	2442	3167	4089	4612
**Frequency of multimorbidity patterns in hub diseases’ associated network (% of the complete multimorbidity network)**	196 064 (68.27%)	128 025 (73.16%)	20 341 (64.39%)	30 832 (72.18%)	31 576 (72.97%)	23 050 (71.80%)	64 403 (65.00%)	9052 (64.24%)	14 526 (60.70%)	16 496 (67.58%)	14 532 (75.40%)
**United Kingdom**											
***N* of health records**	180 497	79 652	9726	15 334	24 255	30 337	100 845	13 801	21 549	31 721	33 774
***N* of diseases and diagnoses**	215, 221 470	215, 101 683	298, 9128	193, 16 377	20 028 601	206, 40 846	187, 119 214	183, 14 845	170, 23 460	182, 36 996	200, 41 846
***N* of inpatients with a single disease (% of the total number of inpatients)**	125 648 (69.61%)	54 840 (68.85%)	7413 (76.22%)	11 002 (71.75%)	16 709 (68.89%)	19 716 (64.99%)	70 808 (70.21%)	10 179 (73.76%)	15 522 (72.03%)	22 293 (70.28%)	22 814 (67.55%)
***N* of inpatients with multimorbidity ≥2 conditions (% of the total number of inpatients)**	54 849 (30.39%)	25 812 (31.15%)	2313 (23.78%)	4332 (28.25%)	7546 (31.11%)	10 621 (35.01%)	30 037 (29.79%)	3622 (26.24%)	6027 (27.97%)	9428 (29.72%)	10 960 (32.45%)
***N* of nodes and edges in the complete multimorbidity network**	215, 467	215, 438	298, 369	193, 262	200, 311	206, 352	187, 377	183, 197	170, 222	182, 291	200, 347
***N* of nodes and edges in the hub diseases network**	10, 13	10, 28	10, 6	10, 15	10, 22	10, 21	10, 16	10, 9	10, 13	10, 20	10, 13
***N* of nodes and edges in hub diseases’ associated network (% of the complete multimorbidity network)**	95 (44.19%), 176 (37.69%)	73 (33.95%), 154 (35.16%)	57 (19.13%), 71 (19.24%)	52 (26.94%), 82 (31.30%)	58 (29.00%), 98 (31.51%)	60 (29.13%), 106 (30.11%)	79 (42.25%), 141 (37.40%)	50 (27.32%), 61 (30.96%)	49 (28.82%), 74 (33.33%)	53 (29.12%), 95 (32.65%)	62 (31.00%), 107 (30.84%)
**Frequency of multimorbidity patterns in the complete multimorbidity network**	50 375	24 180	1597	3563	6513	10 036	25 508	2209	4310	7408	9444
**Frequency of multimorbidity patterns in the hub diseases network**	6967	5226	177	738	1692	2072	2453	166	873	1347	1114
**Frequency of multimorbidity patterns in hub diseases’ associated network (% of the complete multimorbidity network)**	28 014 (55.61%)	13 094 (54.15%)	739 (46.27%)	2131 (59.81%)	4047 (62.14%)	5495 (54.75%)	14 034 (55.02%)	741 (33.54%)	2697 (62.58%)	4128 (55.72%)	5507 (58.31%)

### Study indicators

The disease categories were determined using the 3-character codes of the ICD-10 system. Chinese inpatients had 929 diseases (854 in males, 819 in females), while UK inpatients had 834 diseases (724 in males, 736 in females) (Supplementary_summary: https://docs.google.com/spreadsheets/d/1TE1Txasom_VQqRrP88WqNSdOmp8VWNq3/edit?usp=sharing&ouid=110854432052954855465&rtpof=true&sd=true).

### Definition of multimorbidity and multimorbidity pattern

“Multimorbidity” denotes having 2 or more diseases, and “Complex multimorbidity” signifies 4 or more diseases. “Multimorbidity pattern” describes the co-occurrence of 2 diseases as a disease pair.^3^^,^^6^

### Construction of multimorbidity network

The networks comprised nodes (diseases) and edges (unique multimorbidity patterns). Logistic regression models calculated odds ratios (OR) for each multimorbidity pattern. Selection criteria for network edges included: (1) multimorbidity pattern with OR >1; (2) *P*-value < .05/*N* (with Bonferroni correction, where *N* is the number of patterns with OR >1); (3) multimorbidity pattern with a prevalence >1/10 000 were included to ensure common patterns, while those with lower prevalence were excluded (Supplementary Text S4).

### Three-level multimorbidity networks

**Figure 1.**
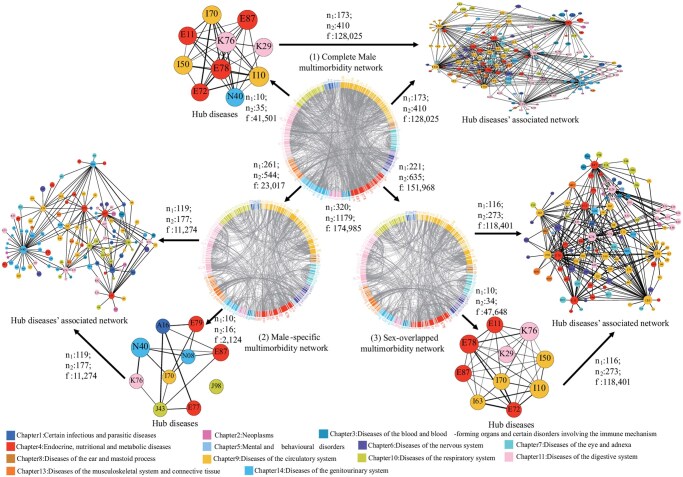
Multimorbidity patterns in Chinese male inpatients. The Chinese male complete multimorbidity network (1), male-specific multimorbidity network (2), and male-overlapped multimorbidity network (multimorbidity patterns shared with females) (3) as well as their Hub diseases and Hub diseases’ associated network. We presented the diseases in ICD-10 codes. Different colors denote different disease systematic chapters in the figure legend. n1: The number of nodes in the corresponding networks. n2: The number of edges in the corresponding networks. f: The total frequency of multimorbidity patterns in the corresponding networks. Node size was based on degree and the thickness of each edge was determined by OR values. Hub disease name with ICD-10 codes (listed by alphabetical order): A16: Respiratory tuberculosis, not confirmed bacteriologically or histologically, E11: Type 2 diabetes mellitus, E72: Other disorders of amino-acid metabolism, E77: Disorders of glycoprotein metabolism, E78: Dyslipidemia, E79: Disorders of purine and pyrimidine metabolism, E87: Other disorders of fluid, electrolyte, and acid-base balance, I10: Essential hypertension, I50: Heart failure, I63: Cerebral infarction, I70: Atherosclerosis, J43: Emphysema, J98: Other respiratory disorders, K29: Gastritis and duodenitis, K76: Other diseases of liver, N08: Glomerular disorders in diseases classified elsewhere, N40: Hyperplasia of prostate.

**Figure 2.**
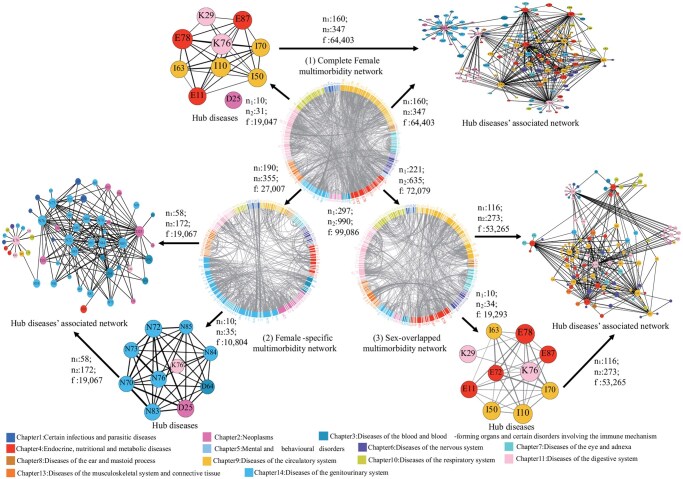
Multimorbidity patterns in Chinese female inpatients. The Chinese female complete multimorbidity network (1), female-specific multimorbidity network (2), and female-overlapped multimorbidity network (multimorbidity patterns shared with males) (3) as well as their Hub diseases and Hub diseases’ associated network. We presented the diseases in ICD-10 codes. Different colors denote different disease systematic chapters in the figure legend. n1: The number of nodes in the corresponding networks. n2: The number of edges in the corresponding networks. f: The total frequency of multimorbidity patterns in the corresponding networks. Node size was based on degree and the thickness of each edge was determined by OR values. Hub disease name with ICD-10 codes (listed by alphabetical order): D25: Leiomyoma of uterus, D64: Other anemias, E11: Type 2 diabetes mellitus, E72: Other disorders of amino-acid metabolism, E78: Dyslipidemia, E79: Disorders of purine and pyrimidine metabolism, E87: Other disorders of fluid, electrolyte, and acid-base balance, I10: Essential hypertension, I50: Heart failure, I63: Cerebral infarction, I70: Atherosclerosis, J43: Emphysema, K29: Gastritis and duodenitis, K76: Other diseases of liver, N70: Salpingitis and oophoritis, N72: Inflammatory disease of cervix uteri, N73: Other female pelvic inflammatory diseases, N76: Other inflammation of vagina and vulva, N83: Noninflammatory disorders of ovary, fallopian tube, and broad ligament, N84: Polyp of female genital tract, N85: Other noninflammatory disorders of cervix uteri.

**Figure 3.**
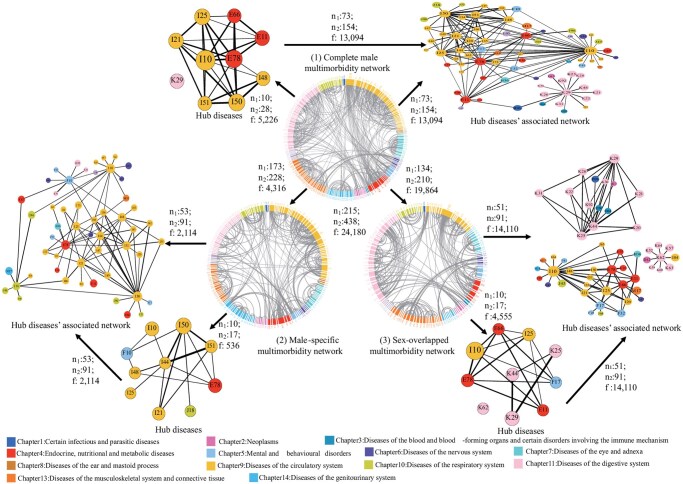
Multimorbidity patterns in the UK male inpatients. The UK male complete multimorbidity network (1), male-specific multimorbidity network (2), and male-overlapped multimorbidity network (multimorbidity patterns shared with females) (3) as well as their Hub diseases and Hub diseases’ associated network. We presented the diseases in ICD-10 codes. Different colors denote different disease systematic chapters in the figure legend. n1: The number of nodes in the corresponding networks. n2: The number of edges in the corresponding networks. f: The total frequency of multimorbidity patterns in the corresponding networks. Node size was based on degree and the thickness of each edge was determined by OR values. Hub disease name with ICD-10 codes (listed by alphabetical order): E11: Type 2 diabetes mellitus, E66: Obesity, E78: Dyslipidemia, F10: Mental and behavioral disorders due to use of alcohol, F17: Mental and behavioral disorders due to use of tobacco, I10: Essential hypertension, I21: Acute myocardial infarction, I25: Chronic ischemic heart disease, I44: Atrioventricular and left bundle-branch block, I48: Atrial fibrillation and flutter, I50: Heart failure, I51: complications and ill-defineddescriptions of heart disease, J18: Pneumonia, organism unspecified, K25:Gastric ulcer, K29: Gastritis and duodenitis, K44: Diaphragmatic hernia, K62: Other diseases of anus and rectum.

**Figure 4.**
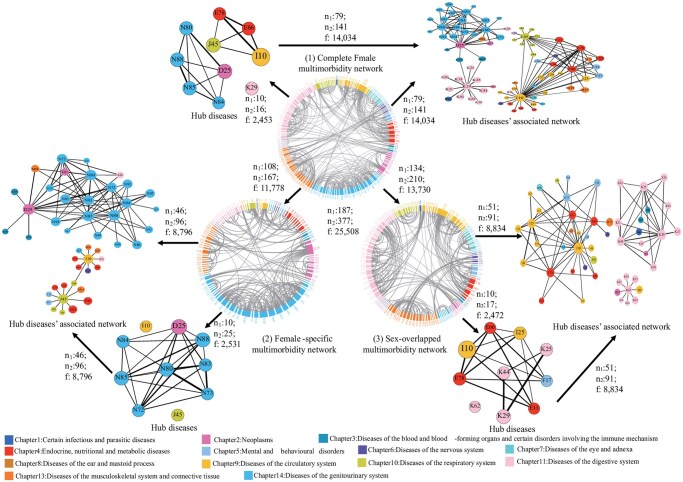
Multimorbidity patterns in the UK female inpatients. The UK female complete multimorbidity network (1), female-specific multimorbidity network (2), and female-overlapped multimorbidity network (multimorbidity patterns shared with males) (3) as well as their Hub diseases and Hub diseases’ associated network. We presented the diseases in ICD-10 codes. Different colors denote different disease systematic chapters in the figure legend. *n*1: The number of nodes in the corresponding networks. *n*2: The number of edges in the corresponding networks. *f*: The total frequency of multimorbidity patterns in the corresponding networks. Node size was based on degree and the thickness of each edge was determined by OR values. Hub disease name with ICD-10 codes (listed by alphabetical order): D25: Leiomyoma of uterus, E11: Type 2 diabetes mellitus, E66: Obesity, E78: Dyslipidemia, F17: Mental and behavioral disorders due to use of tobacco, I10: Essential hypertension, I25: Chronic ischemic heart disease, J45: Asthma, K25: Gastric ulcer, K29: Gastritis and duodenitis, K44: Diaphragmatic hernia, K62: Other diseases of anus and rectum, N72: Inflammatory disease of cervix uteri, N73: Other female pelvic inflammatory diseases, N80: Endometriosis, N83: Noninflammatory disorders of ovary, fallopian tube, and broad ligament, N84: Polyp of female genital tract, N85: Other noninflammatory disorders of cervix uteri, N88: Other noninflammatory disorders of uterus, except cervix.

Supplementary Text S4 showed detailed information on constructing the 3-level multimorbidity networks. The first level involved sex-stratified networks, creating separate “complete multimorbidity network” for males and females in each region (Figures 1-4(1) and Figures S3 and S4 [see online supplementary material for a color version of this figure]; Table 1). The second level entailed a comparative analysis of these networks to identify “sex-specific” (Figures 1-4(2) and Figures S5 and S7 [see online supplementary material for a color version of this figure]; Table S1) and “sex-overlapped” (Figures 1-4(3) and Figures S6 and S8 [see online supplementary material for a color version of this figure]; Table S1) multimorbidity networks based on nonoverlapping and overlapping patterns, revealing similarities and differences in disease interconnections across sex in both regions. The third level, incorporating sex and age stratifications, aimed to identify “sex–age-specific” multimorbidity networks, highlighting how these networks vary with age across different sexes and regions (Figures S9-S24 [see online supplementary material for a color version of this figure]; Table S2). We used degree^32^ (the number of edges a node has) to identify “hub diseases” with the top 10 highest number of unique patterns (edges) in the network. We also included unique patterns associated with hub diseases in “hub diseases’ associated network” (Figures 1-4 and Figures S3-S24 [see online supplementary material for a color version of this figure]).

### Multimorbidity network metric

We analyzed each node using 7 metrics: degree,^32^ maximal clique centrality,^33^ closeness centrality (Clo_Cen),^32^ clustering coefficient (Clu_Coe),^32^ betweenness centrality (Bet_Cen),^32^ pageranks,^34^ and eigencentrality^32^ (Supplementary Text S5). Node size was based on degree, edge thickness on OR values, and ICD-10 chapters determined node colors.

### Statistical analysis

The prevalence of diseases and multimorbidity patterns were calculated, with age measured as mean ± SD. Analyses used R 4.1.0 and Python 2020.1.3, with Cytoscape and Gephi for network analysis. Stacked bar charts, line charts, and violin plots were created using Microsoft Excel and GraphPad Prism 9.3.1.

## Results

### Sociodemographic characteristics of the Chinese and UK inpatients

In Chinese inpatients, average ages were 48.27 (SD ± 5.31) for males and 48.67 (SD ± 5.27) for females, with most aged 40-49 (59.83% males, 56.57% females). UK inpatients had higher averages: 51.82 (SD ± 5.26) for males and 51.30 (SD ± 5.26) for females, with most aged 50-59 (68.54% males, 64.95% females).

### Disease profile in Chinese and UK inpatients by sex and age

In middle-aged inpatients, diagnoses per capita increased with age from 40 to 59 years in China (males: 2.10-2.89, females: 1.86-2.69) and the United Kingdom (males: 1.31-1.58, females: 1.33-1.53). Primary diagnoses were circulatory diseases in Chinese (males: 0.72/person, females: 0.49/person), digestive diseases in British males (0.40/person), and genitourinary diseases in British females (0.39/person) (Tables S3-S6).

### Multimorbidity profile in Chinese and UK inpatients by sex and age

In China, multimorbidity prevalence (57.12%) surpassed single disease diagnosis (42.88%), opposite to the United Kingdom (30.39% multimorbidity, 69.61% single disease). Males exhibited higher multimorbidity prevalence than females in China (58.51% vs 55.33%) and the United Kingdom (31.15% vs 29.79%) (Table 1).

Figure S25 (see online supplementary material for a color version of this figure) showed increasing multimorbidity and decreasing single disease prevalence with age in all groups. Specifically, single disease prevalence dropped, while the prevalence of inpatients with multimorbidity (≥2 diseases) escalated by age. Notably, inpatients with ≥4 diseases (defined as complex multimorbidity^3^) increased to 16.37%-31.07% in Chinese males, 11.75%-26.39% in Chinese females, while in British males it was 1.41%-4.36% and in females 1.61%-3.44% (Table S7). Figure S26 (see online supplementary material for a color version of this figure) showed that in China, both males and females exhibit an apparent increase in disease count with age, particularly in the 50-54 and 55-59 age groups. In contrast, this trend is less pronounced in the UK population, where disease counts remain relatively stable across different age groups.

### Complete multimorbidity networks in the Chinese and UK inpatients by sex

The complete multimorbidity network complexity in Chinese inpatients was significantly higher than in the United Kingdom, with increases of 1.69-fold in males (China: 320 diseases/1179 patterns; United Kingdom: 215 diseases/438 patterns) and 1.63-fold in females (China: 297 diseases/990 patterns; United Kingdom: 187 diseases/377 patterns). The primary hub diseases in both Chinese and British males were circulatory, endocrine/nutritional/metabolic, and digestive diseases. Chinese females had similar hub diseases as males, while British females more often faced genitourinary-associated patterns (Figures 1-4(1)). Notably, hub disease’s associated networks accounted for over 54% of total pattern frequency in all groups, reaching 73.16% (128 025/174 985) in Chinese males and 65.00% (64 403/99 086) in females, compared to 54.15% (13 094/24 180) and 55.02% (14 034/25 508) in UK counterparts, respectively (Table 1; Figures 1-4(1)).

### Multimorbidity networks for overlapping multimorbidity patterns between sexes in Chinese and UK inpatients

We found 635 patterns across 211 diseases overlapped between sexes in China, while 210 patterns across 134 diseases overlapped in the United Kingdom (Figures 1-4(3)).

Notably, hub diseases presented in both sexes in the overlapping network were associated with endocrine/nutritional/metabolic, circulatory, and digestive diseases in China, while in the United Kingdom, also associated with mental and behavioral disorders (Figures 1-4(3)).

### Multimorbidity networks for male-specific multimorbidity patterns in Chinese and UK inpatients

The male-specific multimorbidity network complexity in Chinese inpatients was significantly higher than in the United Kingdom, with increases of 1.39-fold (China: 261 diseases/544 patterns; United Kingdom: 173 diseases/228 patterns) (Figures 1 and 3(2); Table 1).

In the Chinese male-specific multimorbidity network, the hub diseases include metabolic (disorders of fluid/electrolyte/acid-base balance, E87; disorders of purine/pyrimidine metabolism, E79; disorders of glycoprotein metabolism, E77), genitourinary (glomerular disorders in diseases, N08; hyperplasia of prostate, N40), circulatory (atherosclerosis, I70), digestive (liver diseases, K76), infectious (respiratory tuberculosis, A16), and respiratory (respiratory disorders, J98; emphysema, J43) diseases. Conversely, the UK network for males involves circulatory (hypertension, I10; acute myocardial infarction, I21; chronic ischemic heart disease, I25; atrioventricular/left ­bundle-branch block, I44; atrial fibrillation and flutter I48; heart failure, I50; complications and ill-defined descriptions of heart disease, I51), metabolic (dyslipidemia, E78), mental and behavioral (disorders due to alcohol, F10), and respiratory (pneumonia, J18) diseases (Figures 1 and 3(2); Table S8). Multimorbidity patterns covered by its hub diseases’ associated network accounted for 48.98% of the frequency of patterns in China (11 274/23 017) and the same percentage in the United Kingdom (2114/4316) (Figures 1 and 3(2); Table S1).

### Multimorbidity networks for female-specific multimorbidity patterns in Chinese and UK inpatients

The female-specific multimorbidity network complexity in Chinese inpatients was significantly higher than in the United Kingdom, with increases of 1.13-fold (China: 190 diseases/355 patterns; United Kingdom: 108 diseases/167 patterns) (Figures 2 and 4(2); Table 1).

In both China and UK female-specific multimorbidity networks, there were 6 overlapping hub diseases, mainly originating from genitourinary diseases (inflammatory disease of cervix uteri, N72; female pelvic inflammatory diseases, N73; noninflammatory disorders of ovary, fallopian tube and broad ligament, N83; polyp of female genital tract, N84; noninflammatory disorders of uterus, N85), and neoplasms (leiomyoma of uterus, D25). In China, specific hub diseases included other genitourinary diseases (salpingitis/oophoritis, N70; inflammation of vagina and vulva, N76), polyp of female genital tract (D64), and liver diseases (K76). Conversely, in the United Kingdom, specific hub diseases include genitourinary (noninflammatory disorders of cervix uteri, N88; endometriosis, N80), respiratory (asthma, J45), and circulatory (hypertension, I10) diseases (Figures 2 and 4(2); Table S9). Multimorbidity patterns covered by its hub diseases’ associated network accounted for 70.60% of the frequency of patterns in China (19 067/27 007) and 74.68% in the United Kingdom (8796/11 778) (Figures 2 and 4(2); Table S1).

### Multimorbidity networks stratified by age in Chinese and UK male inpatients

Across all age strata (40-44, 45-49, 50-54 years) in China, each complete male-age-specific multimorbidity network was always more complex than in United Kingdom (China: 279/735, 291/808, 298/773, 293/695 diseases/patterns; United Kingdom: 298/369, 193/262, 200/311, 206/352 diseases/patterns) (Figures S17-S20 [see online supplementary material for a color version of this figure]; Table S2).

In Chinese males, hub diseases mainly included circulatory, endocrine/nutritional/metabolic, digestive diseases, of which gastritis/duodenitis (K29), hypertension (I10), dyslipidemia (E78), heart failure (I50), liver diseases (K76), atherosclerosis (I70), fluid/electrolyte/acid-base imbalances (E87) across all age strata. In UK males, hub diseases trends shifted from mental and behavioral disorders (due to tobacco use, F17; due to alcohol use, F10), genitourinary (acute renal failure, N17), and digestive (diaphragmatic hernia, K44) to circulatory and metabolic diseases, of which gastritis/duodenitis (K29), hypertension (I10), dyslipidemia (E78), chronic ischemic heart disease (I25) across all age groups (Figures 1, 3 and Figures S17-S20 [see online supplementary material for a color version of this figure]; Table S10). Whether in China or the United Kingdom, the hub diseases’ associated multimorbidity networks, as a percentage of the corresponding complete multimorbidity networks increased from 64.39% in China and 46.27% in the United Kingdom at ages 40-44, to 71.80% in China and 54.75% in the United Kingdom at ages 55-59 (Table S2).

### Multimorbidity networks stratified by age in Chinese and UK female inpatients

Across all age strata (40-44, 45-49, 50-54 years) in China, each complete female-age-specific multimorbidity network was always more complex than in United Kingdom (China: 238/492, 277/646, 266/640, 256/552 diseases/patterns; United Kingdom: 183/197, 170/222, 182/291, 200/347 diseases/patterns) (Table 1; Figures S9-S16 and S21-S24 [see online supplementary material for a color version of this figure]).

In Chinese females, hub diseases mainly included circulatory, endocrine/nutritional/metabolic, digestive diseases, similar to Chinese males. In UK females, hub diseases, particularly in the 45-55 age group, were mainly concentrated on genitourinary diseases (female pelvic inflammatory diseases, N73; endometriosis, N80, etc.). As age increases, there is a tendency toward circulatory, endocrine/nutritional/metabolic, digestive diseases, and mental/behavioral disorders due to tobacco (F17) (Figures S21-S24 and S27 [see online supplementary material for a color version of this figure]; Table S11). Whether in China or the United Kingdom, the hub diseases’ associated networks, as a percentage of the corresponding complete multimorbidity networks increased from 64.24% in China and 33.54% in the United Kingdom at ages 40-44, to 75.40% in China and 58.31% in the United Kingdom at ages 55-59 (Table 1).

### ICD-10 chapter-associated multimorbidity patterns across sex-specific and sex–age-specific networks in Chinese and British populations.

#### Sex-specific multimorbidity networks

In male-specific multimorbidity networks, both Chinese and British males primarily exhibited circulatory, genitourinary, and endocrine/nutritional/metabolic disease-associated patterns, However, the British males were more focused on ­circulatory-associated patterns, (47.24% vs 36.34% in China), while Chinese males showed broader multimorbidity profile.

**Figure 5.**
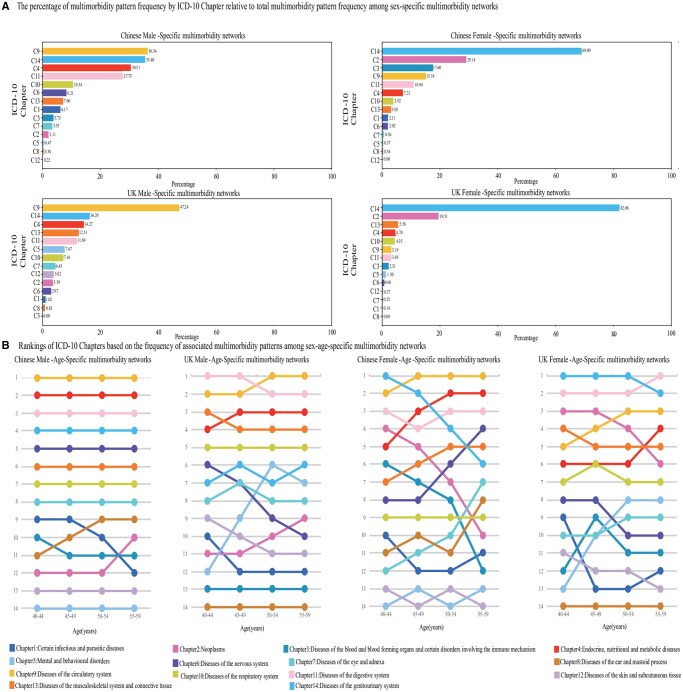
Comparison of multimorbidity pattern frequencies by ICD-10 chapters across sex-specific and sex–age-specific networks in Chinese and British populations. (A) Our study calculated the frequency of multimorbidity patterns associated with each ICD-10 chapter among sex-specific multimorbidity networks in both China and the United Kingdom. Then, we calculated the percentage of multimorbidity pattern frequency by ICD-10 chapter relative to the total multimorbidity pattern frequency among each sex-specific multimorbidity networks. Using these percentages, we ranked the ICD-10 chapters from highest to lowest. The horizontal axis represents the percentage, while the vertical axis lists the ICD-10 chapters. Each bar is colored according to the corresponding ICD-10 chapter, allowing for an easy comparison of chapter frequencies across different populations and sexes. (B) Our study calculated the total frequency of multimorbidity patterns associated with each ICD-10 chapter (Figure S25, see online supplementary material for a color version of this figure). Using these frequencies, we ranked the ICD-10 chapters from highest to lowest, resulting in a final ranking for chapters 1 through 14. This ranking forms the line chart. Taking the Chinese male population as an example, the horizontal axis represents 4 age groups: 40-44 years, 45-49 years, 50-54 years, and 55-59 years. The vertical axis represents the ranking from 1 to 14. Each line is colored according to the corresponding chapter.

In female-specific multimorbidity networks, genitourinary, neoplasm disease-associated patterns dominated in both populations. However, British females showed a concentrated multimorbidity in genitourinary diseases (82.08% vs 69.00% in China), while Chinese females displayed a more varied multimorbidity profile, also with notable frequencies in blood and immune disease-associated patterns (Figure 5A).

#### Sex–age-specific multimorbidity networks

In males, age had minimal impact on chapter rankings. In China, patterns associated with circulatory, endocrine/nutritional/metabolic, digestive, genitourinary, and nervous diseases remained consistent across ages. In the United Kingdom, circulatory, endocrine/nutritional/metabolic disease-associated patterns increased, digestive and musculoskeletal/connective tissue-associated patterns decreased. In particular, mental and behavioral disorder-associated patterns have the largest increase in the United Kingdom.

In females, age impacted chapter rankings more significantly. In China, circulatory, endocrine/nutritional/metabolic, and digestive disease-associated patterns dominated, while genitourinary and neoplasm-associated patterns declined. Notably, nervous and eye/adnexa-associated patterns increased most with age. In the United Kingdom, genitourinary and digestive-associated patterns remained dominant, with circulatory and endocrine/nutritional/metabolic-associated patterns rising and neoplasm-associated patterns declining. In particular, mental and behavioral disorder-associated patterns increased the most in the United Kingdom (Figure 5B and Figure S25 [see online supplementary material for a color version of this figure]).

### The metrics distribution of nodes in Chinese and UK inpatients by age and sex

Hub disease metrics ranked highly among all node-associated metrics for Chinese and UK inpatients by sex and age. Figures S17-S20 (see online supplementary material for a color version of this figure) showed a wider metric distribution among nodes in China, indicating more complex multimorbidity networks compared with the United Kingdom.

## Discussion

Our study compared multimorbidity patterns in middle-aged inpatients by sex and age between China and the United Kingdom. China showed higher multimorbidity prevalence and complexity across all demographics, especially in complex multimorbidity (≥4 diseases), which rose with age in both countries. Males had higher prevalence and complexity than females in both countries. In male-specific networks, Chinese and British males commonly exhibited circulatory, genitourinary, and endocrine/metabolic patterns, with British males more focused on circulatory diseases, while Chinese males had a broader profile. In female-specific networks, genitourinary and neoplasm-­related patterns prevailed. British females concentrated on genitourinary, whereas Chinese females had a more varied profile. Multimorbidity prevalence and network complexity increased with age, altering dominant patterns and hub diseases. In later middle age, males developed more circulatory and endocrine/metabolic-associated patterns. Chinese females saw an increase in nervous system-associated patterns and a decline in genitourinary-associated patterns, while British females experienced rising mental/behavioral-associated patterns.

Our investigation revealed that China had a higher prevalence of multimorbidity than single diseases, unlike the United Kingdom. In China, the higher prevalence is attributed to factors like unequal medical resource allocation,^35^ unrealized comprehensive health insurance coverage,^36^ and limited health awareness,^37^ leading to delayed hospital presentations. Additionally, lifestyle and environmental factors like high-fat diets, insufficient exercise,^38^^,^^39^ air pollution,^40^ and high-stress work^41^ contribute to the early onset of chronic diseases in China’s younger inpatient population.^42^ In contrast, the United Kingdom’s advanced healthcare strategies, including the NHS, focus on early detection and management of diseases, reducing multimorbidity risk.^43^ The United Kingdom’s superior integration of health information technology, particularly electronic health records, facilitates interdisciplinary collaboration and early intervention, potentially lowering multimorbidity rates.^44^

The literature predicts a global increase in complex multimorbidity (4 or more chronic diseases in an individual) over the next 2 decades.^4^ Our study highlighted a pronounced trend in China, where an ageing population significantly contributed to the rise in complex multimorbidity. Patients with complex multimorbidity face challenges like higher morbidity and mortality, increased healthcare demands, complex treatments, and reduced quality of life.^3^^,^^4^ Aging-associated declines in biological and immune functions contribute to chronic disease accumulation, underscoring the need to address complex multimorbidity.^45^ In China, it is more prevalent than in the United Kingdom, exacerbated by factors such as economic and occupational stress, unhealthy lifestyles, delayed healthcare access, and gaps in healthcare infrastructure and early interventions.^46^

Our study uncovered distinct multimorbidity networks in middle-aged individuals in the United Kingdom and China, with clear sex-specific patterns: Females predominantly showed genitourinary disease-associated patterns, while males were more prone to circulatory and endocrine/nutritional/metabolic disorders. These differences are partly due to hormonal changes, with females experiencing a loss of ovarian hormones (estrogen and progesterone) during menopause, leading to an accelerated accumulation of multimorbidity and adverse health outcomes,^47^^,^^48^ while males’ testosterone decline raises cardiovascular diseases and hypertension risk.^49^ Additionally, the prevalence of alcohol-associated comorbidities among British males, attributed to excessive drinking,^50^ highlights the role of cultural and lifestyle factors in shaping sex-specific disease patterns. These findings highlight the importance of understanding sex-specific multimorbidity patterns and the need for tailored healthcare strategies. Analyzing sex-specific multimorbidity networks across countries is valuable for healthcare professionals and policymakers.

The study reveals significant differences in multimorbidity patterns among middle-aged women in China and the United Kingdom. In the United Kingdom, genitourinary-related patterns are consistently higher across all age groups, while in China, it decline with age, and circulatory and metabolic-­related patterns increase, with a more complex disease spectrum. Key factors include as follows. First, the United Kingdom’s healthcare system promotes regular screening and treatment of genitourinary diseases, leading to higher healthcare utilization,^51^ while delayed screening and cultural stigma in China result in underdiagnosis and reluctance to seek care.^52^ Second, in the United Kingdom, alcohol consumption,^50^ high BMI,^53^ and certain sexual behaviors^54^ increase genitourinary disease risk, whereas in China, high salt intake, hypertension, and diabetes contribute to circulatory and metabolic diseases.^55^^,^^56^ Third, genitourinary-related patterns are common in the United Kingdom, partly due to genetic susceptibility, with certain gene loci linked to renal function and genitourinary diseases.^57^ In China, genetic factors influence the transition to metabolic and circulatory diseases, especially in relation to T2D, blood pressure, and lipid metabolism.^58^^,^^59^ As women age in the United Kingdom, the incidence of mental health issue-­related patterns increases, linked to environmental stress and genetic predisposition. The previous study has already proved that mental health disorders are associated with a higher physical multimorbidity burden.^60^ The study showed that certain subtypes of the 5-HTTLPR gene may be more prevalent in Western populations, leading to a higher incidence of psychological disorders.^61^ The NHS implemented early detection and psychological services to facilitate early detection and high diagnostic rates.^62^ In China, the rank of nervous system-related patterns rises with age. The high incidence of metabolic diseases may contribute to neurodegenerative diseases,^60^ exacerbated by circulatory issues and risk factors like high salt intake, hypertension, and hyperglycemia.^63^

This landmark study integrated regional, sex, and age dimensions to analyze multimorbidity patterns across diverse subpopulations. It revealed country-specific multimorbidity networks, influenced by environmental, economic, and cultural factors, and provided a framework for tailored health interventions to advance global health equity. The study also uncovered sex-specific multimorbidity networks, offering insights for targeted prevention, diagnosis, and treatment, contributing to precision medicine. Sex–age-specific networks highlighted the evolution of multimorbidity patterns, guiding age-specific interventions. This approach identifies hub diseases and patterns trends, optimizing public health resource allocation to address varied health needs effectively. This study focuses on multimorbidity in middle-aged populations. As a critical life course period, health status during this phase directly influences chronic disease progression and later-life disease burden. Implementing 3-tiered prevention strategies during this stage can improve subsequent health outcomes, reduce healthcare costs, and alleviate strain on medical systems. Given their role as the core workforce, these measures will also enhance societal productivity and mitigate socioeconomic burdens in later life.

This study has limitations. First, this study uses cross-­sectional data to explore disease correlations but lacks cohort data to examine causal relationships or disease trajectories. Future research should focus on these aspects for deeper insights. Second, variations in data collection methods, and disease numbers between datasets from Shannxi, China, and the UK Biobank pose challenges. However, consistent selection rules for multimorbidity patterns and similar sizes of datasets enable comparisons across sex, age and region. Third, diagnostic inaccuracies may obscure multimorbidity complexity but are unlikely to significantly impact gender-based comparisons. Fourth, focusing solely on hospitalized patients introduces bias by excluding outpatients, potentially affecting the generalizability of findings. Fifth, the study’s geographic restriction to Shannxi Province and the healthier UK Biobank cohort, mainly from England (92%), with 8% from Scotland and Wales, may not represent broader patterns, highlighting the need for more diverse samples in future research. Sixth, the differences in healthcare systems between the 2 countries may impact hospitalization rates. However, identical healthcare systems are rare globally. This study’s exploration of multimorbidity across different systems not only highlights system differences impact of multimorbidity profile but also underscores the role of primary healthcare in multimorbidity development, enhancing its practical significance. Seventh, our study primarily compares Chinese and British populations, which restricts the generalizability of the conclusions. Future research should include data from additional regions to broaden the applicability of multimorbidity research.

This study holds significant translational value, with implications in 3 key areas. First, it examines multimorbidity patterns in the midlife population, a crucial stage in the progression of chronic diseases in older age. Early identification and intervention in these patterns can predict and slow disease progression, improving individual health outcomes and providing early warnings for managing aging populations. Second, the study emphasizes the role of sex and age in shaping multimorbidity patterns, supporting the development of personalized and precise health strategies that can effectively delay the onset of future diseases. Third, through network graph analysis, the study identifies key hub diseases driving multimorbidity, allowing for prioritized, targeted prevention. Addressing these key hub diseases may reduce the disease burden, enhance population health, and alleviate economic burden on healthcare systems.

Our study on multimorbidity in middle-aged inpatients from China and the United Kingdom revealed higher prevalence and complexity in males than females, increasing with age, especially in China. In sex-specific networks, both countries’ females showed strong genitourinary and neoplasm-associated patterns, while males exhibited circulatory, genitourinary, and endocrine/nutritional/metabolic-associated patterns. Chinese multimorbidity profiles were broader. Ageing males developed circulatory and endocrine/nutritional/metabolic-associated patterns, Chinese females shifted toward nervous disease-associated patterns, and UK females saw a rise in mental/behavioral disorder-­associated, with stable genitourinary-­associated patterns.

## Supplementary Material

igaf090_Supplementary_Data

## Data Availability

The data from Shaanxi, China need to be obtained by sending a data request application to the corresponding author’s email and require approval from Shaanxi Provincial People’s Hospital. The data of UK Biobank are available in a public, open access repository. This research has been conducted using the UK Biobank Resource under application number 79244. The UK Biobank data are available on application to the UK Biobank (www.ukbiobank.ac.uk/accessed on August 17, 2022).
