# Supplementary material for: Sex- and age-specific multimorbidity networks in middle-aged inpatients: a network-based comparative study between China and the United Kingdom
Source: Innov Aging. 2025 Aug 18;9(10):igaf090. doi: 10.1093/geroni/igaf090 (PMC12596493; doi:10.1093/geroni/igaf090)
Supplement: igaf090_Supplementary_Data [file igaf090_supplementary_data.docx]

***Innovation in Aging*** **Supplementary Material: Bao et al. Sex- and Age-Specific Multimorbidity Networks in Middle-aged Inpatients: A Network-based Comparative Study Between China and the United Kingdom.**

[Supplementary Text S1. The Content of Chapters under ICD-10 codes. 2](#_Toc180948870)

[Figure S1. The records selection flowchart of the Shannxi, China dataset. 3](#_Toc180948871)

[Supplementary Text S2. The details of Shannxi, China dataset and the overview of the selection of Hospitalization Records from the dataset: (Explanation of Figure S1) 4](#_Toc180948872)

[Figure S2. The records selection flowchart of the UK-Biobank dataset. 7](#_Toc180948873)

[Supplementary Text S3. The details of UK-Biobank dataset and the overview of the selection of Hospitalization Records from the dataset: UK-Biobank Cohort: (Explanation of Figure S2) 8](#_Toc180948874)

[Supplementary Text S4. The construction procedure of a three-level network diagram. 9](#_Toc180948875)

[Figure S3. The selection flowchart of the multimorbidity networks for male and female among Chinese inpatients. 26](#_Toc180948876)

[Figure S4. The selection flowchart of the multimorbidity networks for male and female among British inpatients. 27](#_Toc180948877)

[Figure S5. The selection flowchart of the overlapping multimorbidity networks for male and female inpatients in China. 28](#_Toc180948878)

[Figure S6. The selection flowchart of the nonoverlapping multimorbidity networks for male and female inpatients in China. 29](#_Toc180948879)

[Figure S7. The selection flowchart of the overlapping multimorbidity networks for male and female inpatients in the UK. 30](#_Toc180948880)

[Figure S8. The selection flowchart of the nonoverlapping multimorbidity networks for male and female inpatients in the UK. 31](#_Toc180948881)

[Table S1. Number of nodes, edges and frequency of multimorbidity patterns in the multimorbidity networks stratified by sex in China and the UK. 32](#_Toc180948882)

[Figure S9. The selection flowchart of the multimorbidity networks for 40-44 male and female inpatients in China. 33](#_Toc180948883)

[Figure S10. The selection flowchart of the multimorbidity networks for 45-49 male and female inpatients in China. 34](#_Toc180948884)

[Figure S11. The selection flowchart of the multimorbidity networks for 50-54 male and female inpatients in China. 35](#_Toc180948885)

[Figure S12. The selection flowchart of the multimorbidity networks for 55-59 male and female inpatients in China. 36](#_Toc180948886)

[Figure S13. The selection flowchart of the multimorbidity networks for 40-44 male and female inpatients in the UK. 37](#_Toc180948887)

[Figure S14. The selection flowchart of the multimorbidity networks for 45-49 male and female inpatients in the UK. 38](#_Toc180948888)

[Figure S15. The selection flowchart of the multimorbidity networks for 50-54 male and female inpatients in the UK. 39](#_Toc180948889)

[Figure S16. The selection flowchart of the multimorbidity networks for 55-59 male and female inpatients in the UK. 40](#_Toc180948890)

[Figure S17. Comparisons of complete multimorbidity networks, hub diseases, and hub diseases’ associated network among 40-44 years of Male inpatients between China and the UK. 41](#_Toc180948891)

[Figure S18. Comparisons of complete multimorbidity networks, hub diseases, and hub diseases’ associated network among 45-49 years of Male inpatients between China and the UK. 42](#_Toc180948892)

[Figure S19. Comparisons of complete multimorbidity networks, hub diseases, and hub diseases’ associated network among 50-54 years of Male inpatients between China and UK. 43](#_Toc180948893)

[Figure S20. Comparisons of complete multimorbidity networks, hub diseases, and hub diseases’ associated network among 55-59 years of Male inpatients between China and the UK. 44](#_Toc180948894)

[Figure S21. Comparisons of complete multimorbidity networks, hub diseases, and hub diseases’ associated network among 40-44 years of Female inpatients between China and the UK. 45](#_Toc180948895)

[Figure S22. Comparisons of complete multimorbidity networks, hub diseases, and hub diseases’ associated network among 45-49 years of Female inpatients between China and the UK. 46](#_Toc180948896)

[Figure S23. Comparisons of complete multimorbidity networks, hub diseases, and hub diseases’ associated network among 50-54 years of Female inpatients between China and the UK. 47](#_Toc180948897)

[Figure S24. Comparisons of complete multimorbidity networks, hub diseases, and hub diseases’ associated network among 55-59 years of Female inpatients between China and the UK. 48](#_Toc180948898)

[Supplementary Text S5. The explanation of network metrics. 49](#_Toc180948899)

[S5.1 Degree 49](#_Toc180948900)

[S5.2 Closeness centrality (Clo_Cen) 49](#_Toc180948901)

[S5.3 Clustering coefficient (Clu_Coe) 49](#_Toc180948902)

[S5.4 Between centrality (Bet_Cen) 50](#_Toc180948903)

[S5.5 Pagerank 50](#_Toc180948904)

[S5.5 Eigencentrality 51](#_Toc180948905)

[S5.6 Maximal clique centrality (MCC) 52](#_Toc180948906)

[Table S2. Number of nodes, edges, and frequency of multimorbidity patterns in the multimorbidity networks stratified by age and sex in China and the UK. 53](#_Toc180948907)

[Table S3. The per-capita disease diagnoses of each chapter among each age from 40 to 59 years among Chinese male inpatients. 54](#_Toc180948908)

[Table S4. The per-capita disease diagnoses of each chapter among each age from 40 to 59 years among the UK male inpatients. 55](#_Toc180948909)

[Table S5. The per-capita disease diagnoses of each chapter among each age from 40 to 59 years among Chinese female inpatients. 56](#_Toc180948910)

[Table S6. The per-capita disease diagnoses of each chapter among each age from 40 to 59 years among the UK female inpatients. 57](#_Toc180948911)

[Table S7. The comparison of the percentage of inpatients with various disease conditions among Chinese and the UK inpatients by age and sex](#_Toc180948912)

[Table S8. The hub diseases in the male-specific multimorbidity network among China and the UK. (20 nodes) 59](#_Toc180948913)

[Table S9. The hub diseases in the female-specific multimorbidity network among China and the UK. (14 nodes) 60](#_Toc180948914)

[Table S10. The hub diseases in the 4 subpopulations of male inpatients among China and the UK. (26 nodes) 61](#_Toc180948915)

[Table S11. The hub diseases in the 4 subpopulations of female inpatients among China and the UK. (28 nodes) 62](#_Toc180948916)

[Figure S25. The total frequency of multimorbidity patterns associated with each ICD-10 chapter among sex-age-specific populations in China and the UK. 63](#_Toc180948917)

[Figure S26. Property distribution for all nodes and nodes whose degree ranked top 10 among the male inpatients stratified by four age ranges in China and the UK. 64](#_Toc180948918)

[Figure S27. Property distribution for all nodes and nodes whose degree ranked top 10 among the female inpatients stratified by four age ranges in China and the UK. 65](#_Toc180948919)

[Supplementary summary 66](#_Toc180948920)

[References 67](#_Toc180948921)

**Supplementary Text S1. The Content of Chapters under ICD-10 codes.**

| Chapter | Abbreviation | Content |
| --- | --- | --- |
| Chapter1 | C1 | Certain infectious and parasitic diseases |
| Chapter2 | C2 | Neoplasms |
| Chapter3 | C3 | Diseases of the blood and blood-forming organs and certain disorders involving the immune mechanism |
| Chapter4 | C4 | Endocrine, nutritional and metabolic diseases |
| Chapter5 | C5 | Mental and behavioural disorders |
| Chapter6 | C6 | Diseases of the nervous system |
| Chapter7 | C7 | Diseases of the eye and adnexa |
| Chapter8 | C8 | Diseases of the ear and mastoid process |
| Chapter9 | C9 | Diseases of the circulatory system |
| Chapter10 | C10 | Diseases of the respiratory system |
| Chapter11 | C11 | Diseases of the digestive system |
| Chapter12 | C12 | Diseases of the skin and subcutaneous tissue |
| Chapter13 | C13 | Diseases of the musculoskeletal system and connective tissue |
| Chapter14 | C14 | Diseases of the genitourinary system |
| Chapter15 | C15 | Pregnancy, childbirth and the puerperium |
| Chapter16 | C16 | Certain conditions originating in the perinatal period |
| Chapter17 | C17 | Congenital malformations, deformations and chromosomal abnormalities |
| Chapter18 | C18 | Symptoms, signs and abnormal clinical and laboratory findings, not elsewhere classified |
| Chapter19 | C19 | Injury, poisoning and certain other consequences of external causes |
| Chapter20 | C20 | External causes of morbidity and mortality |
| Chapter21 | C21 | Factors influencing health status and contact with health services |
| Chapter22 | C22 | Codes for special purposes |

#All diagnoses were coded with the International Statistical Classification of Diseases and Related Health Problems 10th revision (ICD-10) ^1^. ICD-10 codes were grouped into 22 disease system chapters. However, our study excluded 15-22 chapters because these diseases are congenital, pregnancy, childbirth, puerperium, perinatal period diseases and symptoms, causes, factors of diseases ^2^.

**Figure S1. The records selection flowchart of the Shannxi, China dataset.**

**
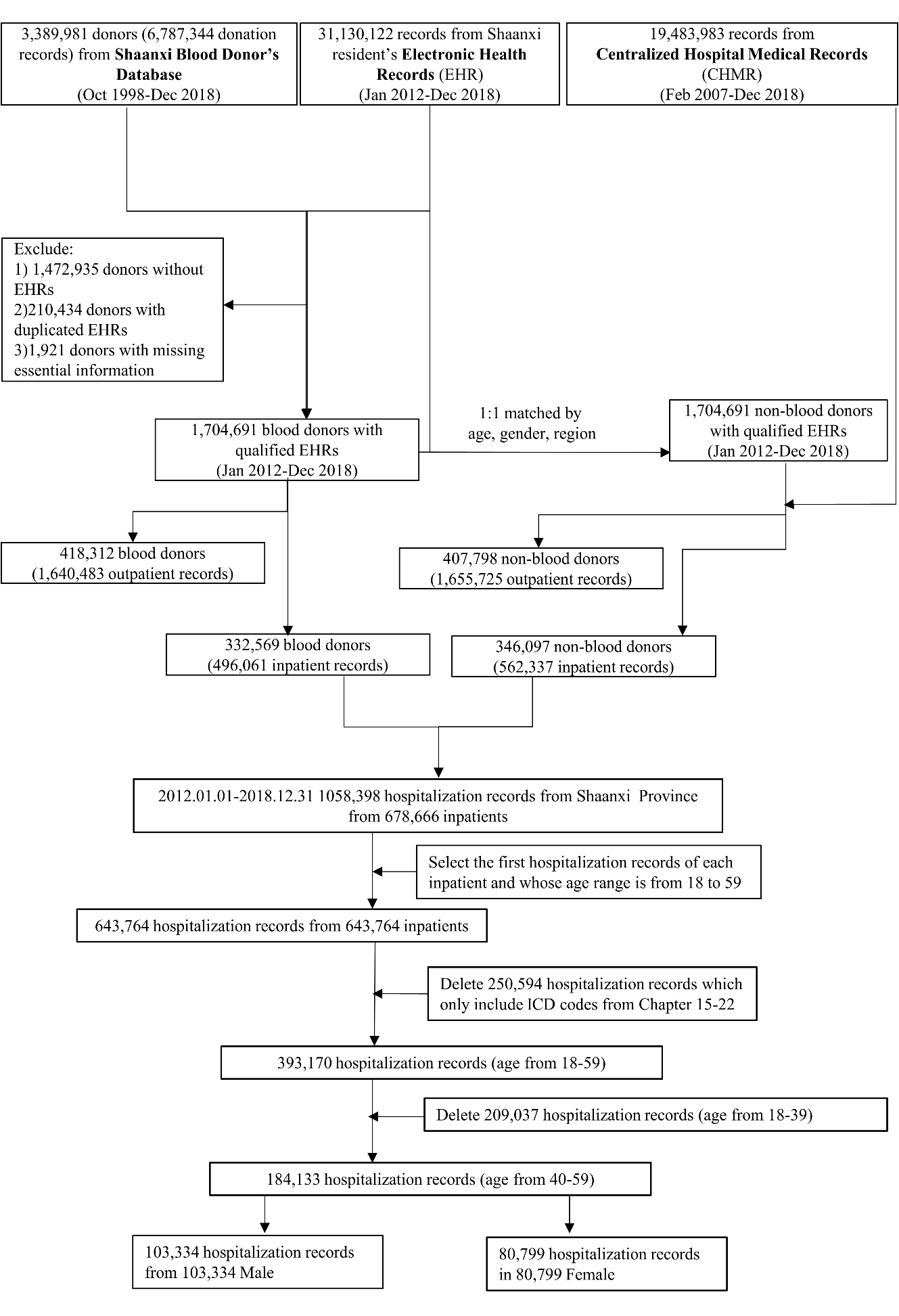
**

**Supplementary Text S2.** **The details of Shannxi, China dataset and the overview of the selection of Hospitalization Records from the dataset: (Explanation of Figure S1)**

The Shaanxi Blood Donor Cohort was established to study the health impacts of blood donation by comparing blood donors and non-donors. We incorporated data from 1.7 million individuals who donated blood between October 1998 and December 2018, drawn from the Shaanxi Blood Donor’s Database. This dataset was then merged with the Electronic Health Records (EHR) of Shaanxi residents. These records are managed by the Shaanxi Provincial Health Planning Policy Evaluation and Information Center. The EHR system, a major digital health initiative launched across China in 2009, was introduced in Shaanxi in 2012.

In 2012, approximately 50% of the 40 million Shaanxi residents were covered by EHR. Its objective was to maintain continuous and trackable medical records for its residents and provide health stakeholders with evidence for disease trends and distribution. Our inclusion criteria were to recruit all the blood donors with an EHR record in the cohort. We, thus, matched the Shaanxi Blood Donor Database with the EHR based on unique resident identification numbers. The linkage resulted in 1,917,046 EHR records matched to the blood donor dataset, and have excluded 1,472,935 donors without EHR. Further, after excluding 210,434 duplications and 1,921 entries that miss essential information, 1,704,691 blood donors were found in files of EHRs and included in this study.

Of the available 31,130,122 records in EHR, we created a non-blood donor cohort with individuals of an identical year of birth, sex, and residency location as the blood donor cohort. The one-to-one matching was conducted through a repeated random selection of individuals without any history of blood donation from the EHR and comparing the three characteristics with those from the blood donor cohort. During the process, the same non-blood donor cannot be selected more than once. The selection process was repeated until all 1,704,691 non-blood donors were selected. The selection of identical years of birth and sex removed the potential confounding effects caused by these demographic characteristics on disease burden. In contrast, selecting identical residency locations ensures that the geographical distributions of diseases between the two cohorts are comparable.

The combined cohort of blood donors and non-blood donors was further linked to the Centralized Hospital Medical Record (CHMR) of Shaanxi Province, also administered by Shaanxi Provincial Health Planning Policy Evaluation and Information Center. In China, hospital outpatient consultations and inpatient admission were compulsorily reportable for state-funded health insurance schemes. As of 2018, CHMR covered 473 public hospitals, 702 private hospitals and 625 community hospitals in Shaanxi. In CHMR, the outpatient records included the date of visit, primary diagnosis and associated complications, and consultation and examination expenses. For inpatients, medical records also include counts of hospital admissions, history of hospitalization, discharge status (discharged or death), department of discharge, and discharge dates or transfer information. All outpatients and inpatients records include patients’ residential identification numbers, which enabled a data linkage with the already integrated blood donor and non-blood donor datasets. As a result, of the 1,704,691 blood donors, a total of 496,061 inpatient records from 332,569 blood donors and 1,640,483 outpatient records from 418,312 blood donors were identified; of the same number of non-blood donors, 562,337 hospital records from 346,097 non-blood donors and 1,655,725 outpatient records from 407,798 non-blood donors were identified. All records’ identification numbers and identifiable information were removed after linkage by the data custodian and blinded from the researchers ^3^.

Our research relies on ICD-10 diagnostic records, which are the clinical gold standard for confirming diseases. Since only inpatient records include complete ICD-10 diagnostic entries, we specifically used these records to study the incidence of multiple diseases, aiming to enhance the accuracy of our research. Initially, we merged 496,061 inpatient records from 332,569 blood donors with 562,337 hospital records from 346,097 non-blood donors, creating a comprehensive hospital record database containing 1,058,398 records involving 678,666 inpatients.

Next, we established baseline data from each patient's first hospital record. Moreover, we excluded records with diagnoses solely categorized under Chapters 15 to 22 of the ICD-10. Given that our study primarily focuses on the middle-aged population, we selected diagnostic records for baseline visits of patients aged from 40 to 59 years. Ultimately, we retained 184,133 records: 103,334 male and 80,799 female.

**Figure S2. The records selection flowchart of the UK-Biobank dataset.**

**
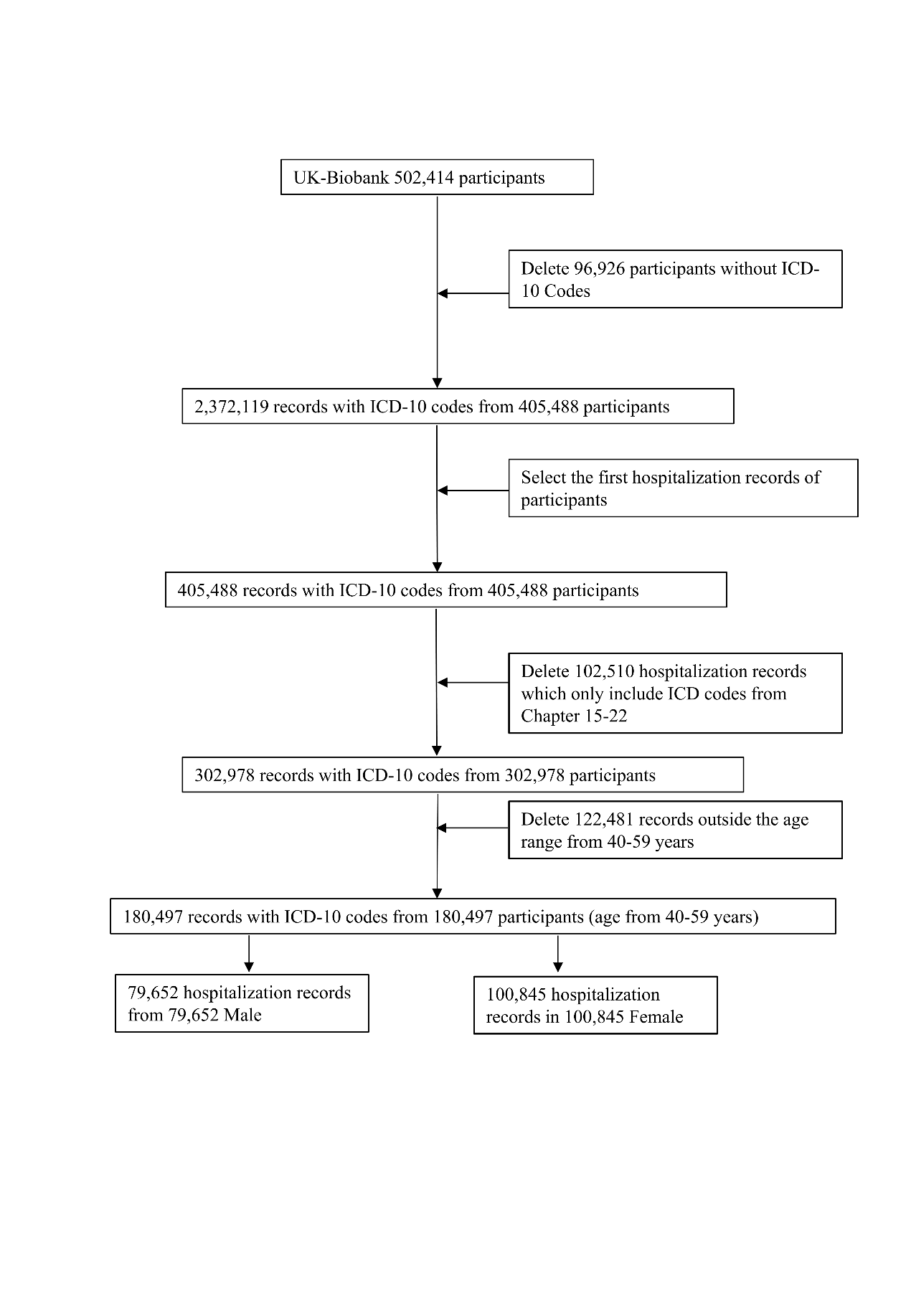
**

**Supplementary Text S3.** **The details of UK-Biobank dataset and the overview of the selection of Hospitalization Records from the dataset: UK-Biobank Cohort: (Explanation of Figure S2)**

The UK Biobank is a large and detailed prospective study with over 502,414 participants, recruited and assessed at 22 centres in England, Wales, and Scotland between 2006 and 2010. Ethical approval was obtained from the North West Multi-Centre Research Ethics Committee, and all participants provided written informed consent. This approval covers the analysis of all data in the present study, including linkage to participants' health records. UK Biobank participants were linked to the UK National Health Service (NHS) Hospital Episode Statistics database for hospital admissions records from March 31, 1992, to May 05, 2021 ^4^. Diseases were coded according to the WHO's International Classification of Diseases 10th Revision (ICD-10).

Initially, our database included 502,414 participants. Among them, 96,926 had no history of hospital admissions. After screening, we identified 40,588 participants with a total of 2,372,119 hospital records. We sorted each individual's records by admission date in descending order and retained the first record as the baseline hospital record, resulting in 40,588 records for 40,588 participants. After excluding records containing only ICD-10 codes from chapters 15-22, we had 302,978 hospital records remaining. Focusing on middle-aged adults (40-59 years), we ultimately retained 180,497 records for our study.

The UK-Biobank (UKB) field codes corresponding to the predictors included in this study are listed in the table below:

| Field Id Code | Description |
| --- | --- |
| X31 | Sex |
| X34 | Year of birth |
| X52 | Month of birth |
| X41270 | Diagnoses-ICD-10 code |
| X41280 | Date of First in-patient diagnosis-ICD10 |

**Supplementary Text S4. The construction procedure of a three-level network diagram.**

## C1, C2: The difference between C1 and C2 is that, while they share the same types of multimorbidity patterns, their manifestations differ. C1 represents patterns specific to Chinese males, with variations in frequency and OR compared to C2. C2 reflects patterns in the Chinese female population. F1, F2: The difference between F1 and F2 is that their manifestations differ while they share the same types of multimorbidity patterns. F1 represents patterns specific to British males, with variations in frequency and OR compared to F2. F2 reflects patterns in the British female population.

**1. Complete** **multimorbidity network (Sex-stratified multimorbidity network)-Object 1**

The construction of the Complete multimorbidity network is based on the corresponding sex-stratified population from the separate database, focusing on four specific demographics: **Chinese male, Chinese female, UK male, and UK female**. We illustrated this process using the example of the Chinese male demographic, comprising 103,334 individuals. Initially, we identified 30,209 multimorbidity patterns within this group (Figure S3). Each pattern was analyzed using logistic regression (adjusting for age), to calculate the odds ratio (OR) and P-value. We retained multimorbidity patterns where the OR was＞1, (indicating that the presence of one disease (Disease A) significantly increases the likelihood of developing another disease (Disease B)), yielding 19,815 multimorbidity patterns. To reduce the likelihood of obtaining false-positive results (Type I errors) when performing multiple statistical tests, these multimorbidity patterns (19,815 multimorbidity patterns with OR>1) were refined using the Bonferroni correction ^5^. This method applied a P-value threshold of < 0.05/19,815 ^6^. Multimorbidity patterns with a P-value below this threshold were retained, resulting in 2,615 patterns remaining. Then, to identify common multimorbidity patterns, we limited the prevalence of patterns to those >1/10,000 in the population (103,334 individuals), resulting in 1,179 multimorbidity patterns being retained. Then we used 1,179 multimorbidity patterns of 320 diseases to constitute the **Chinese Male Complete Multimorbidity network** (Figure 2a_(1), “Supplementary_summary”: Male_Complete_China_Edges/Nodes). The subsequent analysis involved ranking the importance of diseases by the degree metric (the number of edges a node has) from the Complete multimorbidity network, identifying the top ten diseases based on the highest number of unique multimorbidity patterns (edges), termed '**Hub diseases**' (Figure 2a_(1), “Supplementary_summary”: Male_Hub_China_Edges/Nodes). Moreover, we explored the multimorbidity patterns associated specifically with these hub diseases from the Complete multimorbidity network, defining this subset as the **Hub diseases’ associated network** (Figure 2a_(1), “Supplementary_summary”: Male_Hub_associa_China_Edges/Nodes).

Based on the above networks, we identified the corresponding hub diseases in each network and constructed a hub disease-associated network. The following describes the specific conditions of each network. In **Chinese Male Complete multimorbidity network** (**A+C1**, Figure 2a_(1), “Supplementary_summary”: Male_Complete_China_Edges/Male_Complete_China_Nodes), the **‘Hub diseases’** can be seen in Figure 2a_(1)_Hub diseases (“Supplementary_summary”: Male_Hub_China_Edges/Male_Hub_China_Nodes) and **Hub diseases’ associated network** can be seen in Figure 2a_(1)_Hub diseases’ associated network (“Supplementary_summary”: Male_Hub_associa_China_Edges/Male_Hub_associa_China_Nodes). In **Chinese Female Complete multimorbidity network** (**B+C2**, Figure 2b_(1), “Supplementary_summary”: Female_Complete_China_Edges/Nodes), the **‘Hub diseases’** can be seen in Figure 2b_(1)_Hub diseases (“Supplementary_summary”:Female_Hub_China_Edges/Female_Hub_China_Nodes) and **Hub diseases’ associated network** can be seen in Figure 2b_(1)_Hub diseases’ associated network (“Supplementary_summary”: Female_specific_associa_Edges_C/Female_specific_associa_Nodes_C). In **UK Male Complete multimorbidity network** (**D+F1**, Figure 2a_(4), “Supplementary_summary”: Male_Complete_UK_Edges/Male_Complete_UK_Nodes), the **‘Hub diseases’** can be seen in Figure 2a_(4)_Hub diseases (“Supplementary_summary”: Male_Hub_UK_Edges/Male_Hub_UK_Nodes) and **Hub diseases’ associated network** can be seen in Figure 2a_(4)_Hub diseases’ associated network (“Supplementary_summary”: Male_Hub_associa_UK_Edges/Male_Hub_associa_UK_Nodes). In **UK Female Complete multimorbidity network** (**E+F2**, Figure 2b_(4), “Supplementary_summary”: Female_Complete_UK_Edges/Female_Complete_UK_Nodes), the **‘Hub diseases’** can be seen in Figure 2a_(4)_Hub diseases (“Supplementary_summary”: Female_Hub_China_Edges/ Female_Hub_UK_Edges) and **Hub diseases’ associated network** can be seen in Figure 2b_(4)_Hub diseases’ associated network (“Supplementary_summary”: Female_Hub_associa_UK_Edges/Female_Hub_associa_UK_Nodes).

Based on the Venn diagram shown above, the **Chinese Male Complete multimorbidity network** (**A+C1**, Figure 2a_(1), “Supplementary_summary”: Male_Complete_China_Edges/Male_Complete_China_Nodes) consists of two parts: the **Chinese Male-specific multimorbidity network** (**A**, Figure 2a_(2), “Supplementary_summary”: Male_specific_Edges_China/Male_specific_Nodes_China), and the **Chinese Male-overlapped network** (**C1**, Figure 2a_(3), “Supplementary_summary”: Overlap_Male_Edges_China /Overlap_Male_Nodes_China). The **Chinese Female Complete multimorbidity network** (**B+C2**, Figure 2b_(1), “Supplementary_summary”: Female_Complete_China_Edges/Nodes) is composed of the **Chinese Female-specific multimorbidity network** (**B**, Figure 2b_(2), “Supplementary_summary”: Female_specific_Edges_China/Female_specific_Nodes_China), and the **Chinese Female overlapped network** (**C2**, Figure 2b_(3), “Supplementary_summary”: Overlap_Female_Edges_China/Overlap_Female_Nodes_China). The **UK Male Complete multimorbidity network** (**D+F1**, Figure 2a_(4), “Supplementary_summary”: Male_Complete_UK_Edges/Male_Complete_UK_Nodes) consists of the **UK Male-specific multimorbidity network** (**D**, Figure 2a_(5), “Supplementary_summary”: Male_specific_Edges_UK/Male_specific_Nodes_UK), and the **UK Male overlapped network** (**F1**, Figure 2a_(6), “Supplementary_summary”: Overlap_Male_Edges_UK /Overlap_Male_Nodes_UK). Finally, the **UK Female Complete multimorbidity network** (**E+F2**, Figure 2b_(4), “Supplementary_summary”: Female_Complete_UK_Edges/Female_Complete_UK_Nodes) is made up of the **UK Female-specific multimorbidity network** (**E**, Figure 2b_(5), “Supplementary_summary”: Female_specific_Edges_UK/Female_specific_Nodes_UK), and the **UK Female overlapped network** (**F2**, Figure 2b_(6), “Supplementary_summary”: Overlap_Female_Edges_UK/Overlap_Female_Nodes_UK).

**2. Sex-overlapped multimorbidity network-Object 2**

The Sex-overlapped multimorbidity network is derived from various datasets. In this study, four distinct networks are involved: the **Chinese male overlapped multimorbidity network**, the **Chinese female overlapped multimorbidity network**, **the UK male overlapped multimorbidity network, and the UK female overlapped multimorbidity network**. Taking China as an example, the **Chinese male overlapped multimorbidity network** (**C1**, Figure 2a_(3), “Supplementary_summary”: Overlap_Male_Edges_China/Overlap_Male_Nodes_China) is constructed by identifying overlapped multimorbidity patterns from the **Complete multimorbidity network of Chinese males** (1179 multimorbidity patterns) and **Chinese females** (990 multimorbidity patterns). The objective is to obtain multimorbidity patterns overlapped between Chinese males and females to form the corresponding network graph. Consequently, 635 overlapped multimorbidity patterns were identified (Supplementary_summary: Overlap_Male_Edges_China), constituting the **Chinese male overlapped multimorbidity network (C1)**. It is important to note that the Chinese male and female multimorbidity networks differ despite having the same disease combinations in the multimorbidity patterns. The odds ratio (OR) and P-values for the same multimorbidity pattern vary between the two populations. For instance, the OR for the multimorbidity pattern E11/E78 is 3.92 (3.73, 4.12) in males and 4.17 (3.86, 4.50) in females. **The Chinese female overlapped multimorbidity network (C2)** consists of 635 multimorbidity patterns (**C2**, Figure 2b_(3), “Supplementary_summary”: Overlap_Female_Edges_China/ Overlap_Female_Nodes_China).

In the UK population, the method for obtaining the overlapped multimorbidity networks for males and females is the same as that used for the Chinese population. **The UK male overlapped multimorbidity network (F1)** involves 210 multimorbidity patterns (Figure 2a_(6), “Supplementary_summary”: Overlap_Male_Edges_UK / Overlap_Male_Nodes_UK), and **the UK female overlapped multimorbidity network (F2)** also involves 210 multimorbidity patterns (Figure 2b_(6), “Supplementary_summary”: Overlap_Male_Edges_UK/ Overlap_Male_Nodes_UK).

Based on the above networks, we identified the corresponding hub diseases in each network and constructed a hub disease-associated network. The following describes the specific conditions of each network. In **Chinese Male-overlapped network** (**C1**, Figure 2a_(3), “Supplementary_summary”: Overlap_Male_Edges_China /Overlap_Male_Nodes_China), the **‘Hub diseases’** can be seen in Figure 2a_(2)_Hub diseases (“Supplementary_summary”: Overlap_Male_Hub_Edges_China/Overlap_Male_Hub_Nodes_China) and **Hub diseases’ associated network** can be seen in Figure 2a_(3)_Hub diseases’ associated network (“Supplementary_summary”: Overlap_Male_associa_Edges_Ch/Overlap_Male_associa_Nodes_Ch). In **Chinese Female-overlapped network** (**C2**, Figure 2b_(3), “Supplementary_summary”: Overlap_Female_Edges_China / Overlap_Female_Nodes_China), the **‘Hub diseases’** can be seen in Figure 2b_(2)_Hub diseases (“Supplementary_summary”: Overlap_Female_Hub_Edges_China/ Overlap_Female_Hub_Nodes_China) and **Hub diseases’ associated network** can be seen in Figure 2b_(2)_Hub diseases’ associated network (“Supplementary_summary”: Overlap_Female_associa_Edges_Ch/ Overlap_Female_associa_Nodes_Ch). In **UK Male-overlapped network** (**F1**, Figure 2a_(6), “Supplementary_summary”: Overlap_Male_Edges_UK / Overlap_Male_Nodes_UK), the **‘Hub diseases’** can be seen in Figure 2a_(6)_Hub diseases (“Supplementary_summary”: Overlap_Male_Hub_Edges_UK /Overlap_Male_Hub_Nodes_UK) and **Hub diseases’ associated network** can be seen in Figure 2a_(6)_Hub diseases’ associated network (“Supplementary_summary”: Overlap_Male_associa_Edges_UK/ Overlap_Male_associa_Nodes_UK). In **UK Female-overlapped network** (**F2**, Figure 2b_(6), “Supplementary_summary”: Overlap_Male_Edges_UK/ Overlap_Male_Nodes_UK), the **‘Hub diseases’** can be seen in Figure 2b_(6)_Hub diseases (“Supplementary_summary”: Overlap_Female_Edges_UK/Overlap_Female_Nodes_UK) and **Hub diseases’ associated network** can be seen in Figure 2b_(6)_Hub diseases’ associated network (“Supplementary_summary”: Overlap_Female_associa_Edges_UK/Overlap_Female_associa_Nodes_UK).

**3. Sex-specific multimorbidity network-Object 2**

The Sex-specific multimorbidity network is derived from various datasets. In this study, four distinct networks are involved: the Chinese male-specific multimorbidity network, the Chinese female-specific multimorbidity network, the UK male-specific multimorbidity network, and the UK female-specific multimorbidity network.

Taking China as an example, the **Chinese male-specific multimorbidity network** is constructed by identifying 544 non-overlapping multimorbidity patterns from the complete multimorbidity networks of Chinese males (1179 patterns) and females (990 patterns). These patterns are specific to the male network, forming the **Chinese male-specific multimorbidity network (A)** (Supplementary_summary: Male_specific_Edges_China). The Chinese female-specific multimorbidity network is constructed by identifying 355 non-overlapping multimorbidity patterns from the complete multimorbidity networks of Chinese males (1179 patterns) and females (990 patterns). These patterns are specific to the female network, forming the **Chinese female-specific multimorbidity network (B)** (Supplementary_summary: Female_specific_Edges_China).

In the UK population, the method for obtaining the non-overlapped multimorbidity networks for males and females is the same as that used for the Chinese population. The **UK female-specific multimorbidity network (D)** (involves 228 multimorbidity pattern (Supplementary_summary: **Male_specific_Edges_UK**), and the **UK female-specific overlapped multimorbidity network (E)** also involves 167 multimorbidity pattern (Supplementary_summary: **Female_specific_Edges_UK**).

Based on the above networks, we identified the corresponding hub diseases in each network and constructed a hub disease-associated network. The following describes the specific conditions of each network. In **Chinese Male-specific multimorbidity network** (**A**, Figure 2a_(2), “Supplementary_summary”: Male_specific_Edges_China/Male_specific_Nodes_China), the **‘Hub diseases’** can be seen in Figure 2a_(2)_Hub diseases (“Supplementary_summary”: Male_specific_Hub_Edges_China/ Male_specific_Hub_Nodes_China) and **Hub diseases’ associated network** can be seen in Figure 2a_(2)_Hub diseases’ associated network (“Supplementary_summary”: Male_specific_associa_Edges_Ch/Male_specific_associa_Nodes_Ch). In **Chinese Female-specific multimorbidity network** (**B**, Figure 2b_(2), “Supplementary_summary”: Female_specific_Edges_China/ Female_specific_Nodes_China), the **‘Hub diseases’** can be seen in Figure 2b_(2)_Hub diseases (“Supplementary_summary”: Female_specific_Hub_Edges_China/ Female_specific_Hub_Nodes_China) and **Hub diseases’ associated network** can be seen in Figure 2b_(2)_Hub diseases’ associated network (“Supplementary_summary”: Female_specific_associa_Edges_C/ Female_specific_associa_Nodes_C). In **UK Male-specific multimorbidity network** (**D**, Figure 2a_(5), “Supplementary_summary”: Male_specific_ Edges_UK /Male_specific_Nodes_UK), the **‘Hub diseases’** can be seen in Figure 2a_(5)_Hub diseases (“Supplementary_summary”: Male_specific_associa_Edges_UK/Male_specific_associa_Nodes_UK) and **Hub diseases’ associated network** can be seen in Figure 2a_(5)_Hub diseases’ associated network (“Supplementary_summary”: Male_specific_associa_Edges_UK / Male_specific_associa_Nodes_UK). In **UK Female-specific multimorbidity network** (**E**, Figure 2b_(5), “Supplementary_summary”: Female_specific_Edges_UK/ Female_specific_Nodes_UK), the **‘Hub diseases’** can be seen in Figure 2b_(5)_Hub diseases (“Supplementary_summary”: Female_specific_Hub_Edges_UK/Female_specific_Hub_Nodes_UK) and **Hub diseases’ associated network** can be seen in Figure 2b_(5)_Hub diseases’ associated network (“Supplementary_summary”: Female_specific_associa_Edges_UK/Female_specific_associa_Nodes_U).

**4. Sex-Age-specific multimorbidity network-Object 3**

We divided the population into subgroups based on age (40-44, 45-49, 50-54, 55-59) and sex (male and female). For each subgroup, we constructed a complete multimorbidity network. For example, in the male population of China (total n=103,334), the subgroups are as follows: 40-44 years (n=30,167), 45-49 years (n=31,655), 50-54 years (n=25,402), and 55-59 years (n=16,110). Focusing on the 40-44 age group (n=30,167), we constructed the corresponding multimorbidity network. Initially, we identified 13,727 multimorbidity patterns within this group. Each pattern was analyzed using logistic regression, to calculate the odds ratio (OR) and P-value. We retained multimorbidity patterns where the OR was＞1, (indicating that the presence of one disease (Disease A) significantly increases the likelihood of developing another disease (Disease B)), yielding 10,180 multimorbidity patterns. To reduce the likelihood of obtaining false-positive results (Type I errors) when performing multiple statistical tests, these multimorbidity patterns were refined using the Bonferroni correction ^5^. This method targeted a P-value threshold of < 0.05/10,180, resulting in 1,305 multimorbidity patterns remaining. We defined multimorbidity patterns with a prevalence > 1/10,000 of the entire population (30,167 individuals) as common multimorbidity patterns. This criterion reduced the number of multimorbidity patterns to 735, involving 279 diseases (The selection flowchart can be seen in Figure S6), which constituted the **Chinese 40-44 Male Complete Multimorbidity network (**Figure S17**)**. Using the same selection criteria, we constructed the **Chinese 45-49 Male Complete Multimorbidity network**, including 808 multimorbidity patterns and 291 diseases (Figure S7, Figure S18); the **Chinese 50-54 Male Complete Multimorbidity network**, including 773 multimorbidity patterns and 298 diseases (Figure S8, Figure S19); the **Chinese 55-59 Male Complete Multimorbidity network**, including 695 multimorbidity patterns and 293 diseases (Figure S9, Figure S20); **Chinese 40-44 Female Complete Multimorbidity network,** including 492 multimorbidity patterns and 238 diseases (Figure S6, Figure S21); **Chinese 45-49 Female Complete Multimorbidity network**, including 646 multimorbidity patterns and 277 diseases (Figure S7, Figure S22); the **Chinese 50-54 Female Complete Multimorbidity network**, including 640 multimorbidity patterns and 266 diseases (Figure S8, Figure S23); the **Chinese 55-59 Female Complete Multimorbidity network**, including 552 multimorbidity patterns and 256 diseases (Figure S9, Figure S24).

In the UK, using the same selection criteria, we constructed the **UK 40-44 Male Complete Multimorbidity network,** including 369 multimorbidity patterns and 298 diseases (Figure S13, Figure S17); **UK 45-49 Male Complete Multimorbidity network**, including 262 multimorbidity patterns and 193 diseases (Figure S14, Figure S18); the **UK 50-54 Male Complete Multimorbidity network**, including 311 multimorbidity patterns and 200 diseases (Figure S15, Figure S19); the **UK 55-59 Male Complete Multimorbidity network**, including 352 multimorbidity patterns and 206 diseases (Figure S16, Figure S20); **UK 40-44 Female Complete Multimorbidity network,** including 197 multimorbidity patterns and 183 diseases (Figure S13, Figure S21); **UK 45-49 Female Complete Multimorbidity network**, including 222 multimorbidity patterns and 170 diseases (Figure S14, Figure S22); the **UK 50-54 Female Complete Multimorbidity network**, including 291 multimorbidity patterns and 182 diseases (Figure S15, Figure S23); the **UK 55-59 Female Complete Multimorbidity network**, including 347 multimorbidity patterns and 200 diseases (Figure S16, Figure S24).

Based on the above networks, we identified the corresponding hub diseases in each network and constructed a hub disease-associated network. The following describes the specific conditions of each network. In **Chinese 40-44 Male Complete Multimorbidity network (**Figure S17_(1), “Supplementary_summary”: Comp_40-44Male_Edges_China /Comp_40-44Male_Nodes_China **),** the **‘Hub diseases’** can be seen in Figure S17_(2)_Hub diseases (“Supplementary_summary”: Comp_40-44Male_Hub_Edges_China/Comp_40-44Male_Hub_Nodes_China) and **Hub diseases’ associated network** can be seen in Figure S17_(3)_Hub diseases’ associated network (“Supplementary_summary”: Comp_40-44Male_associa_Edges_Ch/Comp_40-44Male_associa_Nodes_Ch). In **Chinese 45-49 Male Complete Multimorbidity network (**Figure S18_(1), “Supplementary_summary”: Comp_45-49Male_Edges_China /Comp_45-49Male_Nodes_China **),** the **‘Hub diseases’** can be seen in Figure S18_(2)_Hub diseases (“Supplementary_summary”: Comp_45-49Male_Hub_Edges_China/Comp_45-49Male_Hub_Nodes_China) and **Hub diseases’ associated network** can be seen in Figure S18_(3)_Hub diseases’ associated network (“Supplementary_summary”: Comp_45-49Male_associa_Edges_Ch/Comp_45-49Male_associa_Nodes_Ch). In **Chinese 50-54 Male Complete Multimorbidity network (**Figure S19_(1), “Supplementary_summary”: Comp_50-54Male_Edges_China /Comp_50-54Male_Nodes_China**),** the **‘Hub diseases’** can be seen in Figure S19_(2)_Hub diseases (“Supplementary_summary”: Comp_50-54Male_Hub_Edges_China/Comp_50-54Male_Hub_Nodes_China) and **Hub diseases’ associated network** can be seen in Figure S19_(3)_Hub diseases’ associated network (“Supplementary_summary”: Comp_50-54Male_associa_Edges_Ch/Comp_50-54Male_associa_Nodes_Ch). In **Chinese 55-59 Male Complete Multimorbidity network (**Figure S20_(1), “Supplementary_summary”: Comp_55-59Male_Edges_China /Comp_55-59Male_Nodes_China**),** the **‘Hub diseases’** can be seen in Figure S20_(2)_Hub diseases (“Supplementary_summary”: Comp_55-59Male_Hub_Edges_China/Comp_55-59Male_Hub_Nodes_China) and **Hub diseases’ associated network** can be seen in Figure S20_(3)_Hub diseases’ associated network (“Supplementary_summary”: Comp_55-59Male_associa_Edges_Ch/Comp_55-59Male_associa_Nodes_Ch).

In **Chinese 40-44 Female Complete Multimorbidity network (**Figure S21_(1), “Supplementary_summary”: Comp_40-44Female_Edges_China /Comp_40-44Female_Nodes_China **),** the **‘Hub diseases’** can be seen in Figure S21_(2)_Hub diseases (“Supplementary_summary”: Comp_40-44Female_Hub_Edges_China/Comp_40-44Female_Hub_Nodes_China) and **Hub diseases’ associated network** can be seen in Figure S21_(3)_Hub diseases’ associated network (“Supplementary_summary”: Comp_40-44Female_associa_Edges_Ch/Comp_40-44Female_associa_Nodes_Ch). In **Chinese 45-49 Female Complete Multimorbidity network (**Figure S22_(1), “Supplementary_summary”: Comp_45-49Female_Edges_China /Comp_45-49Female_Nodes_China **),** the **‘Hub diseases’** can be seen in Figure S22_(2)_Hub diseases (“Supplementary_summary”: Comp_45-49Female_Hub_Edges_China/Comp_45-49Female_Hub_Nodes_China) and **Hub diseases’ associated network** can be seen in Figure S22_(3)_Hub diseases’ associated network (“Supplementary_summary”: Comp_45-49Female_associa_Edges_Ch/Comp_45-49Female_associa_Nodes_Ch). In **Chinese 50-54 Female Complete Multimorbidity network (**Figure S23_(1), “Supplementary_summary”: Comp_50-54Female_Edges_China /Comp_50-54Female_Nodes_China**),** the **‘Hub diseases’** can be seen in Figure S23_(2)_Hub diseases (“Supplementary_summary”: Comp_50-54Female_Hub_Edges_China/Comp_50-54Female_Hub_Nodes_China) and **Hub diseases’ associated network** can be seen in Figure S23_(3)_Hub diseases’ associated network (“Supplementary_summary”: Comp_50-54Female_associa_Edges_Ch/Comp_50-54Female_associa_Nodes_Ch). In **Chinese 55-59 Female Complete Multimorbidity network (**Figure S24_(1), “Supplementary_summary”: Comp_55-59Female_Edges_China /Comp_55-59Female_Nodes_China**),** the **‘Hub diseases’** can be seen in Figure S24_(2)_Hub diseases (“Supplementary_summary”: Comp_55-59Female_Hub_Edges_China/Comp_55-59Female_Hub_Nodes_China) and **Hub diseases’ associated network** can be seen in Figure S24_(3)_Hub diseases’ associated network (“Supplementary_summary”: Comp_55-59Female_associa_Edges_Ch/Comp_55-59Female_associa_Nodes_Ch).

In **UK 40-44 Male Complete Multimorbidity network (**Figure S17_(4), “Supplementary_summary”: Comp_40-44Male_Edges_UK /Comp_40-44Male_Nodes_UK**),** the **‘Hub diseases’** can be seen in Figure S17_(5)_Hub diseases (“Supplementary_summary”: Comp_40-44Male_Hub_Edges_UK/Comp_40-44Male_Hub_Nodes_UK) and **Hub diseases’ associated network** can be seen in Figure S17_(6)_Hub diseases’ associated network (“Supplementary_summary”: Comp_40-44Male_associa_Edges_UK/Comp_40-44Male_associa_Nodes_UK). In **UK 45-49 Male Complete Multimorbidity network (**Figure S18_(4), “Supplementary_summary”: Comp_45-49Male_Edges_UK /Comp_45-49Male_Nodes_UK **),** the **‘Hub diseases’** can be seen in Figure S18_(5)_Hub diseases (“Supplementary_summary”: Comp_45-49Male_Hub_Edges_UK/Comp_45-49Male_Hub_Nodes_UK) and **Hub diseases’ associated network** can be seen in Figure S18_(6)_Hub diseases’ associated network (“Supplementary_summary”: Comp_45-49Male_associa_Edges_UK/Comp_45-49Male_associa_Nodes_UK). In **UK 50-54 Male Complete Multimorbidity network (**Figure S19_(4), “Supplementary_summary”: Comp_50-54Male_Edges_UK /Comp_50-54Male_Nodes_UK**),** the **‘Hub diseases’** can be seen in Figure S19_(5)_Hub diseases (“Supplementary_summary”: Comp_50-54Male_Hub_Edges_UK/Comp_50-54Male_Hub_Nodes_UK) and **Hub diseases’ associated network** can be seen in Figure S19_(6)_Hub diseases’ associated network (“Supplementary_summary”: Comp_50-54Male_associa_Edges_UK /Comp_50-54Male_associa_Nodes_UK). In **UK 55-59 Male Complete Multimorbidity network (**Figure S20_(4), “Supplementary_summary”: Comp_55-59Male_Edges_UK /Comp_55-59Male_Nodes_UK**),** the **‘Hub diseases’** can be seen in Figure S20_(5)_Hub diseases (“Supplementary_summary”: Comp_55-59Male_Hub_Edges_UK/Comp_55-59Male_Hub_Nodes_UK) and **Hub diseases’ associated network** can be seen in Figure S20_(6)_Hub diseases’ associated network (“Supplementary_summary”: Comp_55-59Male_associa_Edges_ UK /Comp_55-59Male_associa_Nodes_UK).

In **UK 40-44 Female Complete Multimorbidity network (**Figure S21_(4), “Supplementary_summary”: Comp_40-44Female_Edges_UK /Comp_40-44Female_Nodes_UK**),** the **‘Hub diseases’** can be seen in Figure S21_(5)_Hub diseases (“Supplementary_summary”: Comp_40-44Female_Hub_Edges_UK/Comp_40-44Female_Hub_Nodes_UK) and **Hub diseases’ associated network** can be seen in Figure S21_(6)_Hub diseases’ associated network (“Supplementary_summary”: Comp_40-44Female_associa_Edges_Ch/Comp_40-44Female_associa_Nodes_Ch). In **UK 45-49 Female Complete Multimorbidity network (**Figure S22_(4), “Supplementary_summary”: Comp_45-49Female_Edges_UK /Comp_45-49Female_Nodes_UK **),** the **‘Hub diseases’** can be seen in Figure S22_(5)_Hub diseases (“Supplementary_summary”: Comp_45-49Female_Hub_Edges_UK/Comp_45-49Female_Hub_Nodes_UK) and **Hub diseases’ associated network** can be seen in Figure S22_(6)_Hub diseases’ associated network (“Supplementary_summary”: Comp_45-49Female_associa_Edges_Ch/Comp_45-49Female_associa_Nodes_Ch). In **UK 50-54 Female Complete Multimorbidity network (**Figure S23_(4), “Supplementary_summary”: Comp_50-54Female_Edges_UK /Comp_50-54Female_Nodes_UK**),** the **‘Hub diseases’** can be seen in Figure S23_(5)_Hub diseases (“Supplementary_summary”: Comp_50-54Female_Hub_Edges_UK/Comp_50-54Female_Hub_Nodes_UK) and **Hub diseases’ associated network** can be seen in Figure S23_(6)_Hub diseases’ associated network (“Supplementary_summary”: Comp_50-54Female_associa_Edges_Ch/Comp_50-54Female_associa_Nodes_Ch). In **UK 55-59 Female Complete Multimorbidity network (**Figure S24_(4), “Supplementary_summary”: Comp_55-59Female_Edges_UK /Comp_55-59Female_Nodes_UK**),** the **‘Hub diseases’** can be seen in Figure S24_(5)_Hub diseases (“Supplementary_summary”: Comp_55-59Female_Hub_Edges_UK/Comp_55-59Female_Hub_Nodes_UK) and **Hub diseases’ associated network** can be seen in Figure S24_(6)_Hub diseases’ associated network (“Supplementary_summary”: Comp_55-59Female_associa_Edges_Ch/Comp_55-59Female_associa_Nodes_Ch).

**Figure S3. The selection flowchart of the multimorbidity networks for male and female among Chinese inpatients.**

****##‘multimorbidity pairs’ means ‘multimorbidity patterns’; ‘hub diseases related diseases’ multimorbidity network’ means ‘hub diseases associated network’

**Figure S4. The selection flowchart of the multimorbidity networks for male and female among British inpatients.**

##‘multimorbidity pairs’ means ‘multimorbidity patterns’; ‘hub diseases related diseases’ multimorbidity network’ means ‘hub diseases associated network’

**Figure S5. The selection flowchart of the overlapping multimorbidity networks for male and female inpatients in China.**

##‘multimorbidity pairs’ means ‘multimorbidity patterns’; ‘hub diseases related diseases’ multimorbidity network’ means ‘hub diseases associated network’

**Figure S6. The selection flowchart of the nonoverlapping multimorbidity networks for male and female inpatients in China.**

##‘multimorbidity pairs’ means ‘multimorbidity patterns’; ‘hub diseases related diseases’ multimorbidity network’ means ‘hub diseases associated network’

**Figure S7. The selection flowchart of the overlapping multimorbidity networks for male and female inpatients in the UK.**

##‘multimorbidity pairs’ means ‘multimorbidity patterns’; ‘hub diseases related diseases’ multimorbidity network’ means ‘hub diseases associated network’

**Figure S8. The selection flowchart of the nonoverlapping multimorbidity networks for male and female inpatients in the UK.**

##‘multimorbidity pairs’ means ‘multimorbidity patterns’; ‘hub diseases related diseases’ multimorbidity network’ means ‘hub diseases associated network’

**Table S1. Number of nodes, edges and frequency of multimorbidity patterns in the multimorbidity networks stratified by sex in China and the UK.**

| Multimorbidity networks | China | | | | | | UK | | | | | |
| --- | --- | --- | --- | --- | --- | --- | --- | --- | --- | --- | --- | --- |
|  | Male | | | Female | | | Male | | | Female | | |
|  | Nodes (%) | Edges (%) | Fre* (%) | Nodes (%) | Edges (%) | Fre* (%) | Nodes (%) | Edges (%) | Fre* (%) | Nodes (%) | Edges (%) | Fre* (%) |
| Complete multimorbidity network | 320 | 1, 179 | 174, 985 | 297 | 990 | 99, 086 | 215 | 438 | 24, 180 | 187 | 377 | 25, 508 |
| Complete hub diseases’ network (Percentage^1^) | 10 (3.13) | 35 (2.97) | 41, 501 (23.72) | 10 (3.37) | 31 (3.13) | 19, 047 (19.22) | 10 (4.65) | 28 (6.39) | 5, 226 (21.61) | 10 (5.35) | 16 (4.24) | 2, 453 (9.62) |
| Complete hub diseases’ associated network (Percentage^2^) | 173 (54.06) | 410 (34.78) | 128, 025 (73.16) | 160 (53.87) | 347 (35.05) | 64, 403 (65.00) | 73 (33.95) | 154 (35.16) | 13, 094 (54.15) | 79 (42.25) | 141 (37.40) | 14,034 (55.02) |
| Overlapped multimorbidity network | 221 | 635 | 151, 968 | 221 | 635 | 72, 079 | 134 | 210 | 19, 864 | 134 | 210 | 13,730 |
| Overlapped hub diseases’ network (Percentage^3^) | 10 (4.52) | 34 (5.35) | 47, 648 (31.35) | 10 (4.52) | 34 (5.35) | 19, 293 (26.77) | 10 (7.46) | 17 (8.10) | 4, 555 (22.93) | 10 (7.46) | 17 (8.10) | 2,472 (18.00) |
| Overlapped hub diseases’ associated network (Percentage^4^) | 116 (52.49) | 273 (42.99) | 118, 401 (77.91) | 116 (52.94) | 273 (42.99) | 53, 265 (73.90) | 51 (38.06) | 91 (43.33) | 14, 110 (71.03) | 51 (38.06) | 91 (43.33) | 8,834 (64.34) |
| Sex-specific multimorbidity network | 261 | 544 | 23, 017 | 190 | 355 | 27, 007 | 173 | 228 | 4, 316 | 108 | 167 | 11,778 |
| Sex-specific hub diseases’ network (Percentage^5^) | 10 (3.83) | 16 (2.94) | 2,124 (9.23) | 10 (5.26) | 35 (9.86) | 10, 804 (40.00) | 10 (5.78) | 17 (7.46) | 536 (12.42) | 10 (9.26) | 25 (14.97) | 2,531 (21.49) |
| Sex-specific hub diseases’ associated network (Percentage^6^) | 119 (45.59) | 177 (32.54) | 11, 274 (48.98) | 58 (30.53) | 172 (48.45) | 19, 067 (70.60) | 53 (30.64) | 91 (39.91) | 2, 114 (48.98) | 46 (42.59) | 96 (57.49) | 8,796 (74.68) |

## Fre*: Frequency

1: The number of nodes/edges and frequency of multimorbidity patterns in the complete hub diseases network accounts for the complete network.

2: The number of nodes/edges and frequency of multimorbidity patterns in the complete hub diseases’ associated network accounts for the complete network.

3: The number of nodes/edges and frequency of multimorbidity patterns in the common hub diseases network accounts for the overlapped network.

4: The number of nodes/edges and frequency of multimorbidity patterns in the common hub diseases’ associated network accounts for the overlapped network.

5: The number of nodes/edges and frequency of multimorbidity patterns in the sex-specific hub diseases network accounts for the sex-specific network.

6: The number of nodes/edges and frequency of multimorbidity patterns in the sex-specific hub diseases’ associated network accounts for the sex-specific network.

**Figure S9. The selection flowchart of the multimorbidity networks for 40-44 male and female inpatients in China.**

##‘multimorbidity pairs’ means ‘multimorbidity patterns’; ‘hub diseases related diseases’ multimorbidity network’ means ‘hub diseases associated network’

**Figure S10. The selection flowchart of the multimorbidity networks for 45-49 male and female inpatients in China.**

**
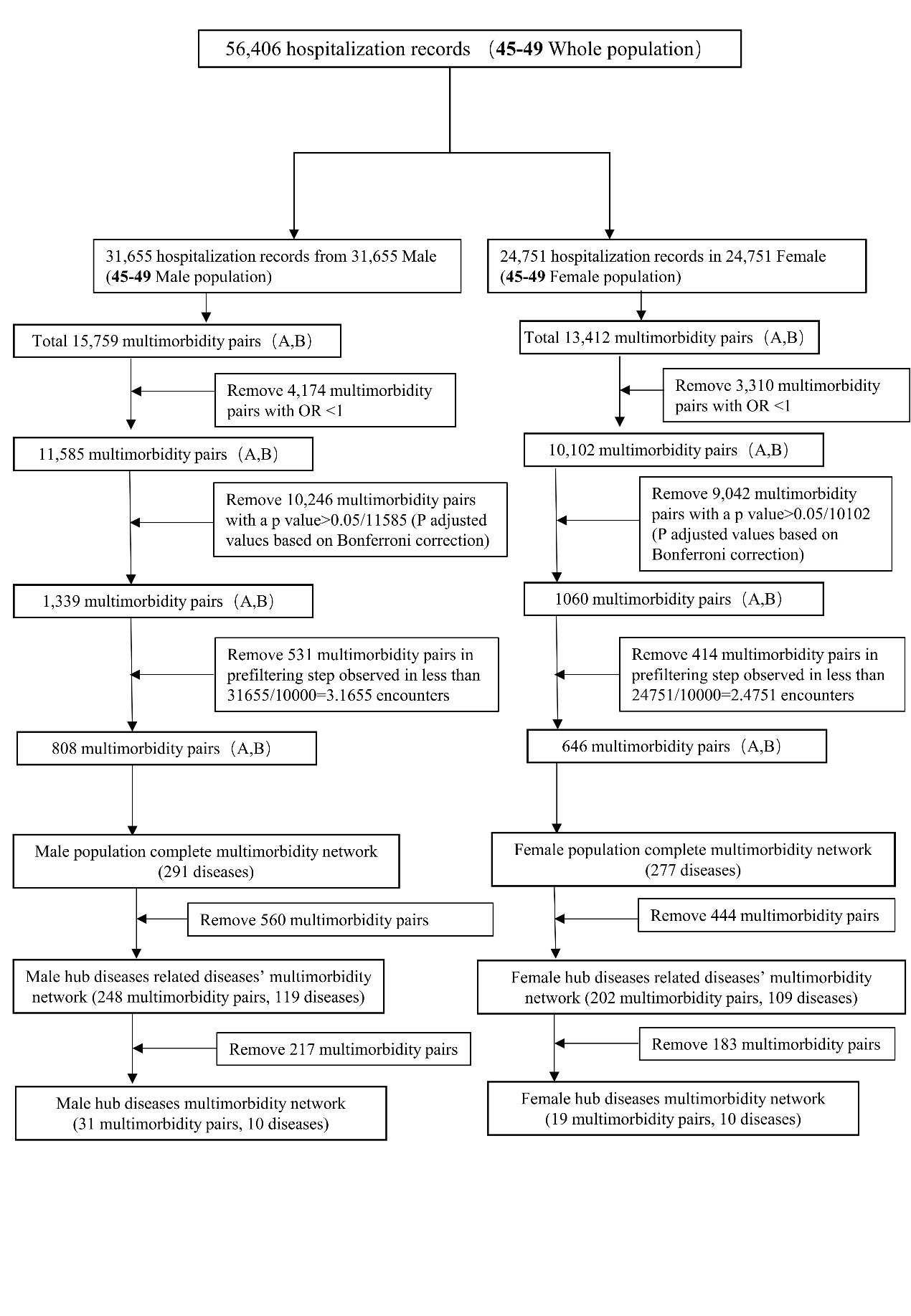
**

##‘multimorbidity pairs’ means ‘multimorbidity patterns’; ‘hub diseases related diseases’ multimorbidity network’ means ‘hub diseases associated network’

**Figure S11. The selection flowchart of the multimorbidity networks for 50-54 male and**

**female inpatients in China.**

**
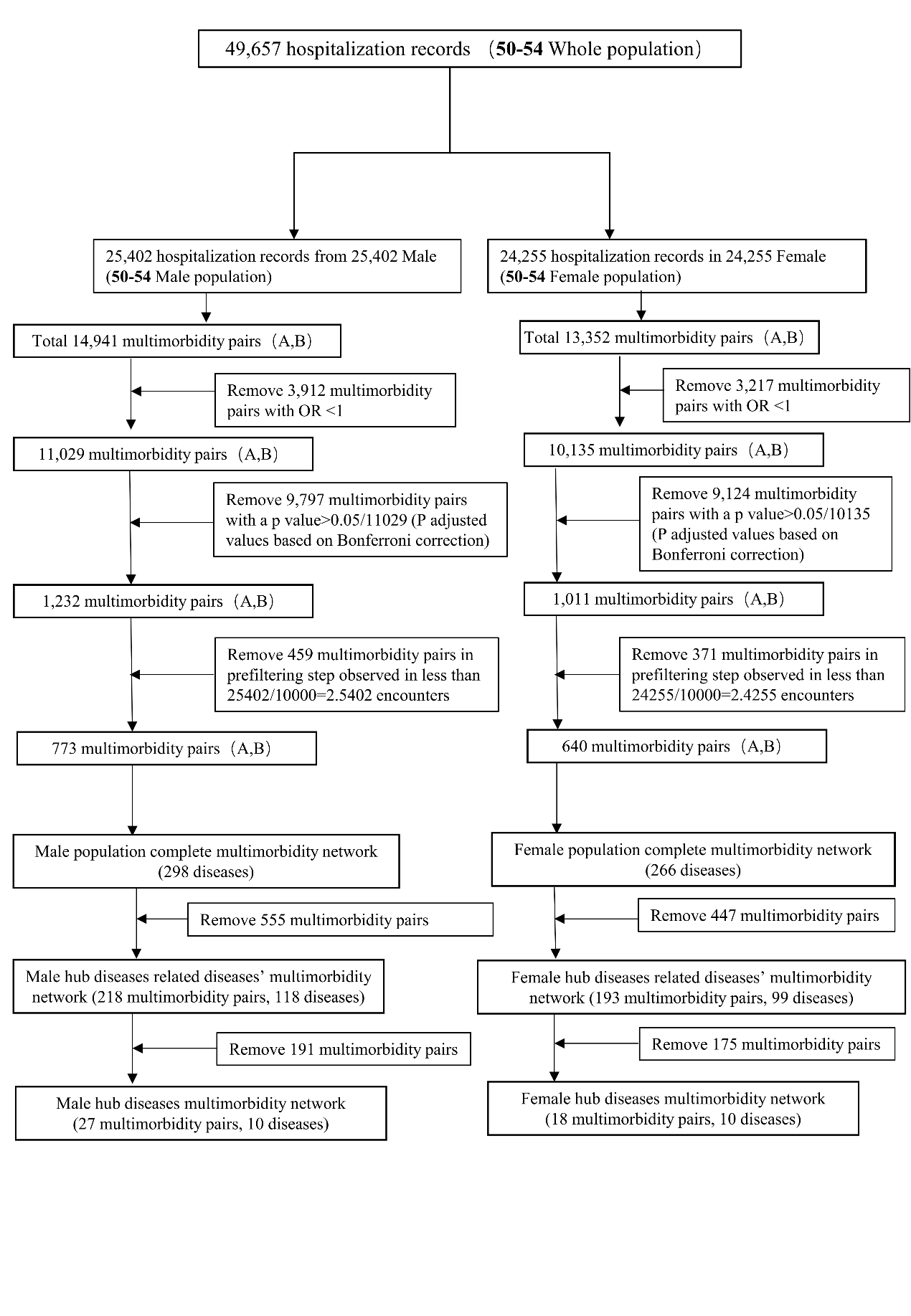
**

##‘multimorbidity pairs’ means ‘multimorbidity patterns’; ‘hub diseases related diseases’ multimorbidity network’ means ‘hub diseases associated network’

**Figure S12. The selection flowchart of the multimorbidity networks for 55-59 male and female inpatients in China.**

**
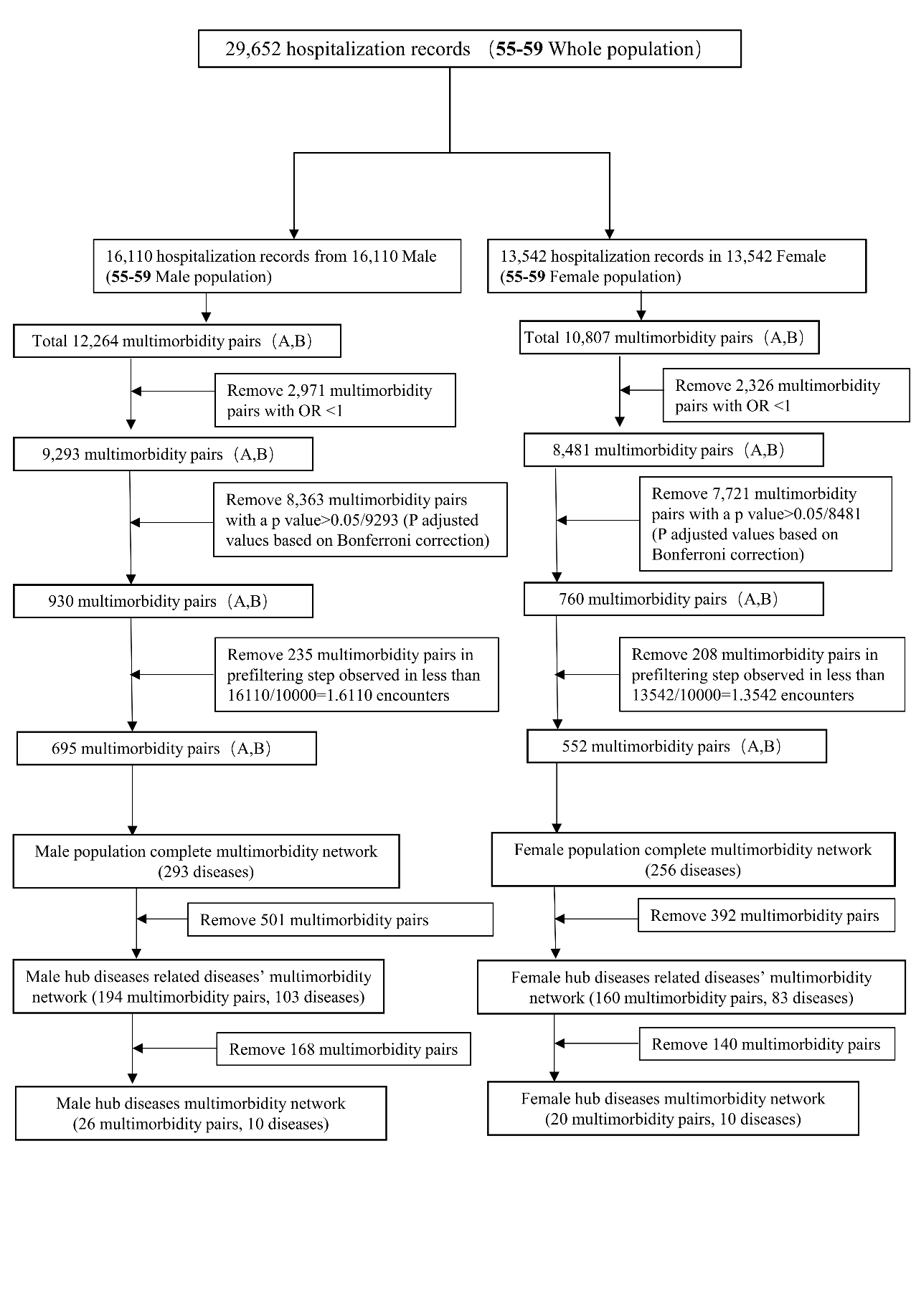
**

##‘multimorbidity pairs’ means ‘multimorbidity patterns’; ‘hub diseases related diseases’ multimorbidity network’ means ‘hub diseases associated network’

**Figure S13. The selection flowchart of the multimorbidity networks for 40-44 male and female inpatients in the UK.**

**
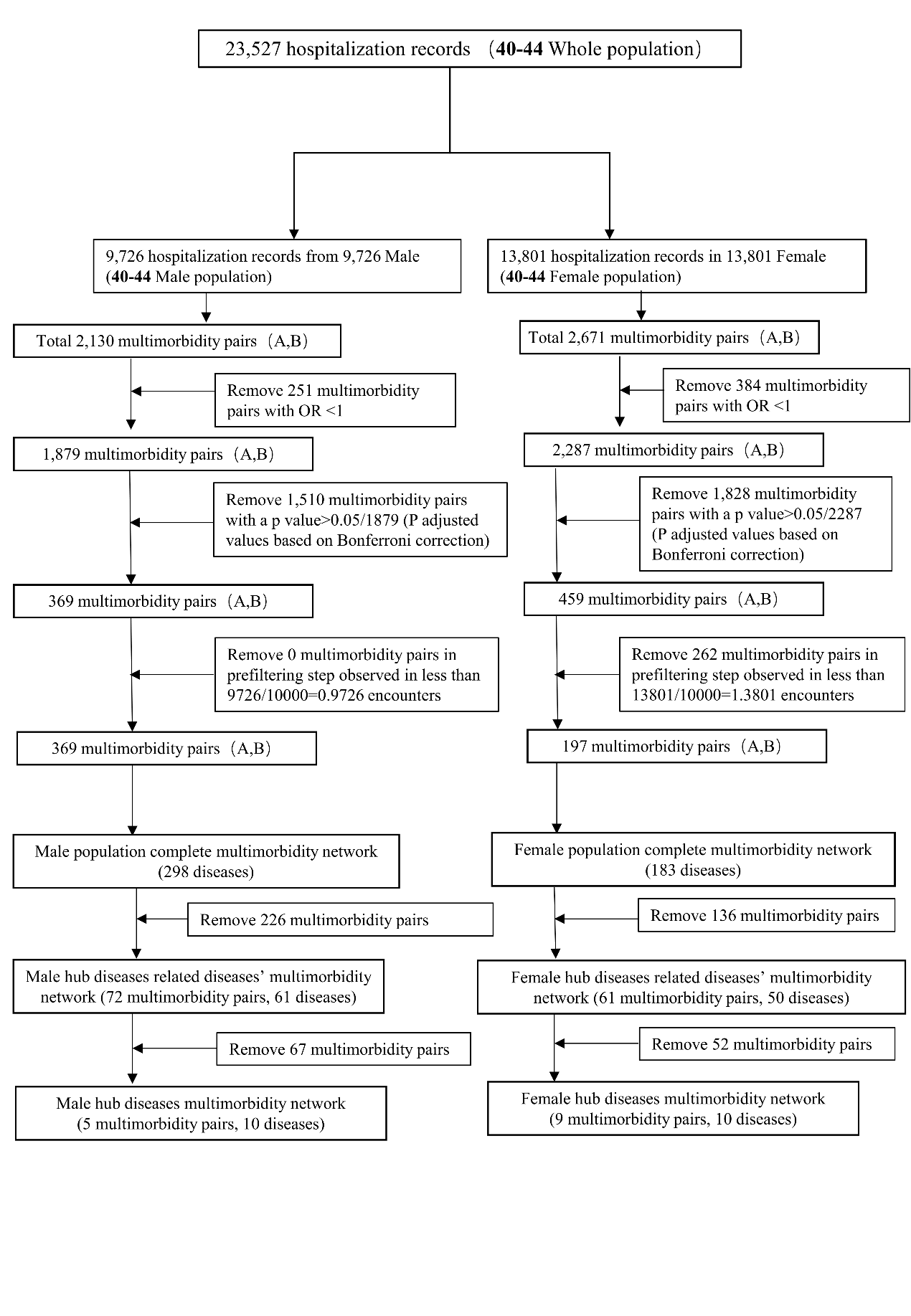
**

##‘multimorbidity pairs’ means ‘multimorbidity patterns’; ‘hub diseases related diseases’ multimorbidity network’ means ‘hub diseases associated network’

**Figure S14. The selection flowchart of the multimorbidity networks for 45-49 male and female inpatients in the UK.**

**
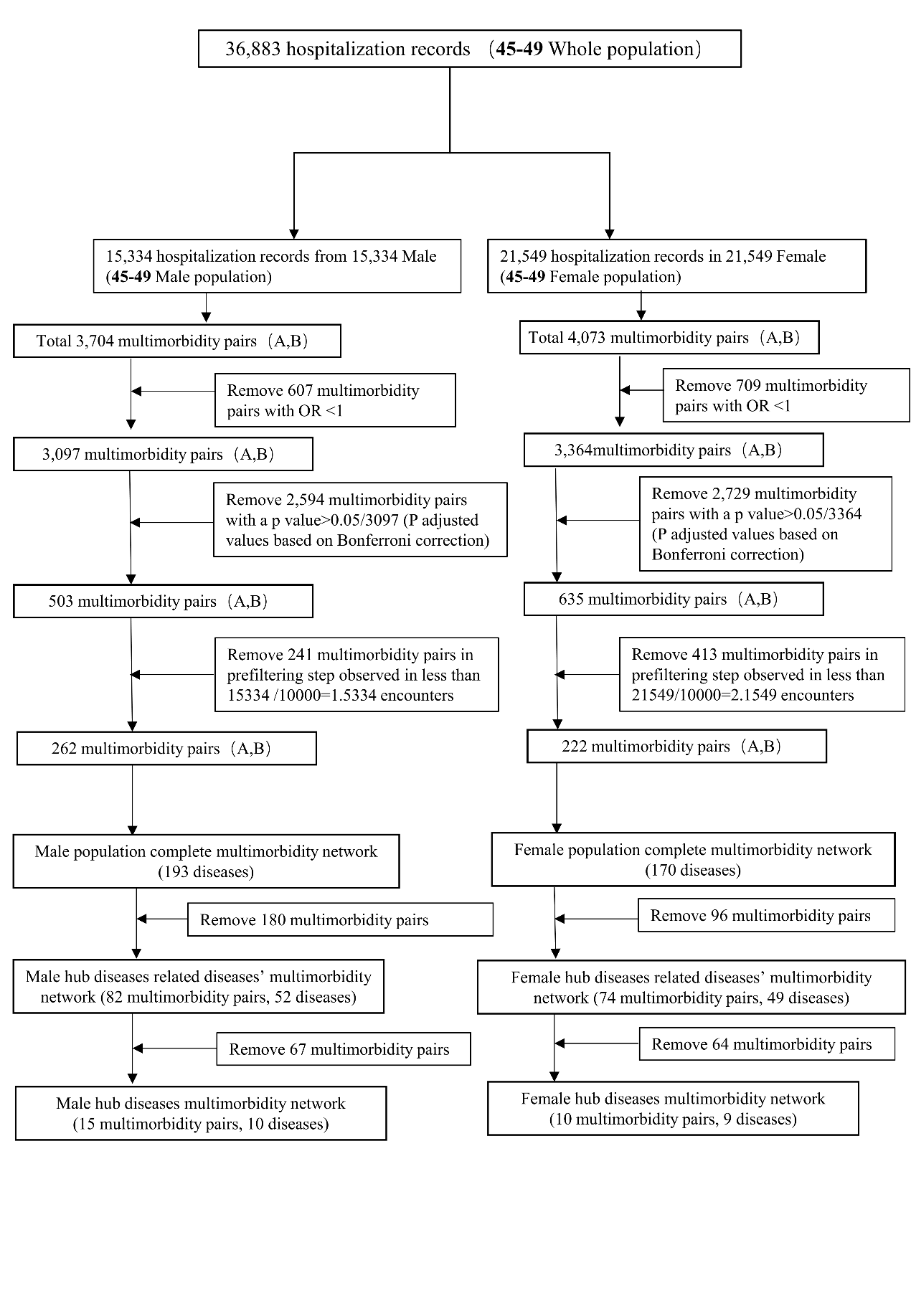
**

##‘multimorbidity pairs’ means ‘multimorbidity patterns’; ‘hub diseases related diseases’ multimorbidity network’ means ‘hub diseases associated network’

**Figure S15. The selection flowchart of the multimorbidity networks for 50-54 male and female inpatients in the UK.**

**
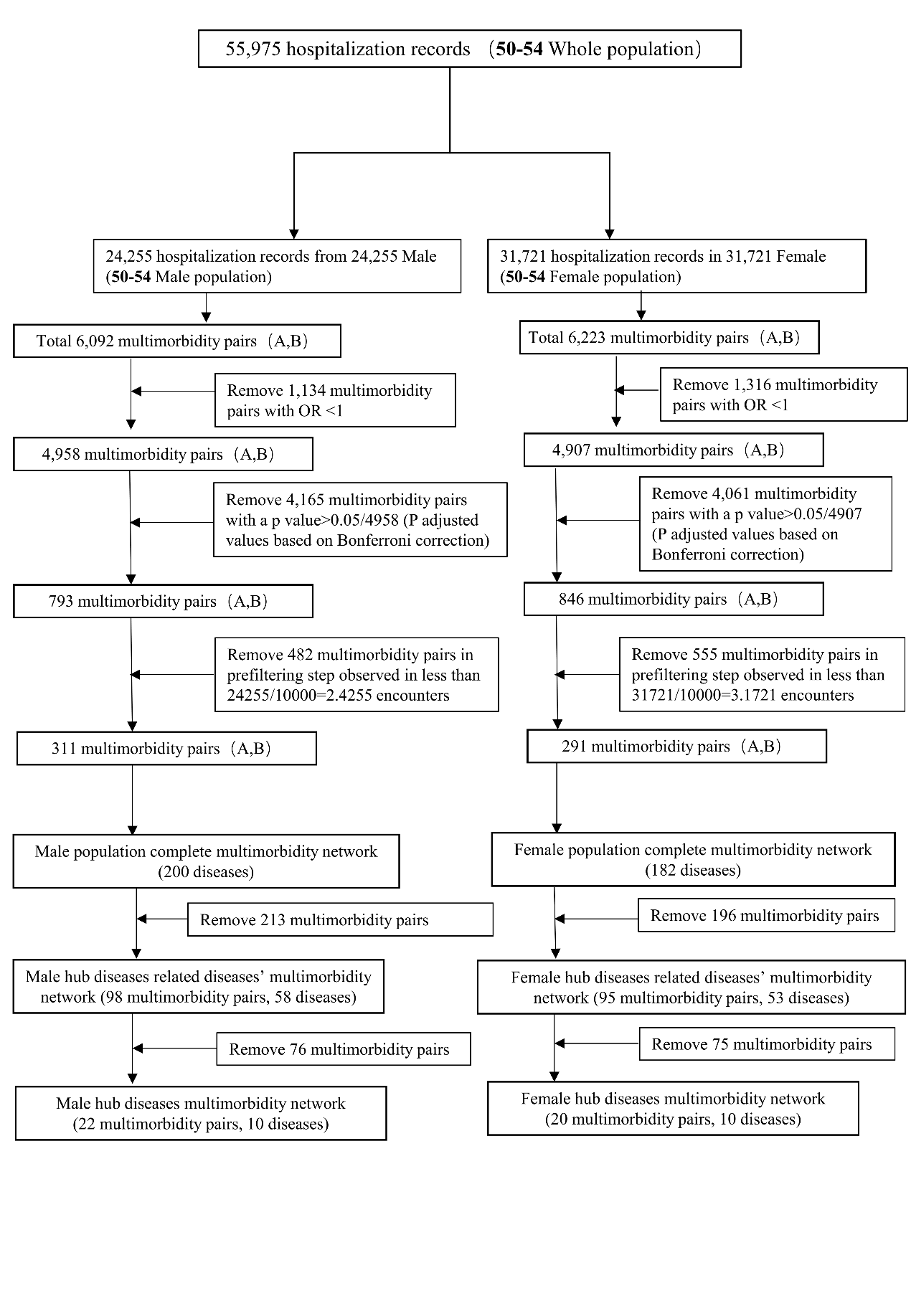
**

##‘multimorbidity pairs’ means ‘multimorbidity patterns’; ‘hub diseases related diseases’ multimorbidity network’ means ‘hub diseases associated network’

**Figure S16. The selection flowchart of the multimorbidity networks for 55-59 male and female inpatients in the UK.**

**
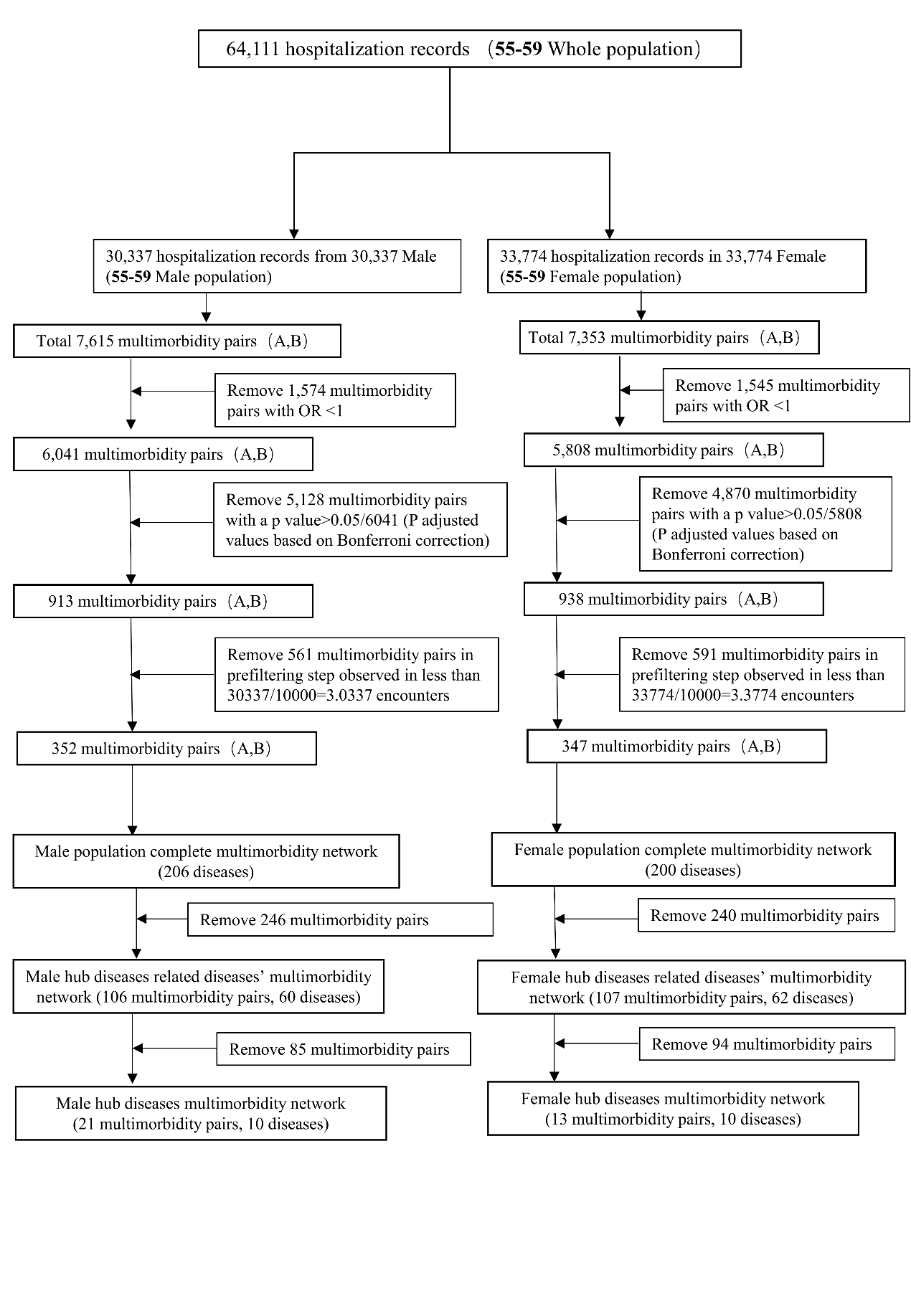
**

##‘multimorbidity pairs’ means ‘multimorbidity patterns’; ‘hub diseases related diseases’ multimorbidity network’ means ‘hub diseases associated network’

**Figure S17. Comparisons of complete multimorbidity networks, hub diseases, and hub diseases’ associated network among 40-44 years of Male inpatients between China and the UK.**

**Figure S18. Comparisons of complete multimorbidity networks, hub diseases, and hub diseases’ associated network among 45-49 years of Male inpatients between China and the UK.**

**Figure S19. Comparisons of complete multimorbidity networks, hub diseases, and hub diseases’ associated network among 50-54 years of Male inpatients between China and UK.**

**Figure S20. Comparisons of complete multimorbidity networks, hub diseases, and hub diseases’ associated network among 55-59 years of Male inpatients between China and the UK.**

**Figure S21. Comparisons of complete multimorbidity networks, hub diseases, and hub diseases’ associated network among 40-44 years of Female inpatients between China and the UK.**

**Figure S22. Comparisons of complete multimorbidity networks, hub diseases, and hub diseases’ associated network among 45-49 years of Female inpatients between China and the UK.**

**Figure S23. Comparisons of complete multimorbidity networks, hub diseases, and hub diseases’ associated network among 50-54 years of Female inpatients between China and the UK.**

**Figure S24. Comparisons of complete multimorbidity networks, hub diseases, and hub diseases’ associated network among 55-59 years of Female inpatients between China and the UK.**

**Supplementary Text S5. The explanation of network metrics.**

**S5.1 Degree**

The degree of a node in an undirected graph is the number of connections or edges the node has to other nodes^7^.

For node *i* in an undirected network, its degree *ki* is defined as:

$$\boldsymbol{k}i=\sum_{j=1}^{N} Aij$$

where A_ij_ represents the adjacency matrix element (1 if nodes *i* and *j* are connected, 0 otherwise), and N is the total number of nodes.

**S5.2 Closeness centrality (Clo_Cen)**

Clo_Cen indicates nodes that can communicate quickly with other nodes of the network. Let G = (V, E) be an undirected graph. Then, the centrality is defined as:

$$\boldsymbol{Cclo}\left( \boldsymbol{i} \right)=\frac{\mathbf{1}}{\sum_{\boldsymbol{t}\in\boldsymbol{V}}^{|\boldsymbol{V}|} \boldsymbol{dist}(\boldsymbol{i}, \boldsymbol{j})}$$

where *dist(i, j)* denotes the distance or else the shortest path *p* between the nodes *i* and *j* ^8^.

**S5.3 Clustering coefficient (Clu_Coe)**

The clustering coefficient assesses the connectivity in a node’s neighborhood: a node has a high clustering coefficient if its neighbors tend to be directly connected with each other. The coefficient is fundamental to assessing the small-world property, and it can be interpreted as an index of the redundancy of a node. A triangle is a subgraph of three nodes all connected to each other. It can be conceived of as a direct connection of a node j with a node q, given by (j, q), plus an indirect connection that travels through another node, i, given by (j, i, q). The local clustering coefficient was initially defined by Watts and Strogatz for unweighted networks as the number of connections among the neighbors of a focal node over the maximum possible number of such connections,

$$\boldsymbol{C}_{\boldsymbol{i}, \boldsymbol{w}}=\frac{\sum_{\boldsymbol{j}, \boldsymbol{q}} (\boldsymbol{a}(\boldsymbol{j}, \boldsymbol{i})\boldsymbol{a}(\boldsymbol{i}, \boldsymbol{q})\boldsymbol{a}(\boldsymbol{j}, \boldsymbol{q})}{\boldsymbol{k}_{\boldsymbol{i}}(\boldsymbol{k}_{\boldsymbol{i}}-\mathbf{1})}$$

where
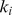
 is the degree of node *i*. The clustering coefficient can be equivalently conceived of as the number of triangles in the neighborhood of a focal node (*t_i_*), normalized by the maximum possible number of such triangles, and it can be interpreted as a measure of how much a focal node *i* is redundant in establishing connections in its neighborhood ^9^.

**S5.4 Between centrality (Bet_Cen)**

*Betweenness Centrality* shows that nodes which are intermediate between neighbors rank higher. Without these nodes, there would be no way for two neighbors to communicate with each other. Thus, *betweenness centrality* shows important nodes that lie on a high proportion of paths between other nodes in the network. For distinct nodes *i, j, w* ∈ *V*(*G*), let *σ_ij_* be the total number of shortest paths between *i* and *j* and *σ_ij_*(*w*) be the number of shortest paths from *i* to *j* that pass through *w*. Moreover, for *w* ∈ *V*(*G*), let *V* (*i*) denote the set of all ordered pairs, (*i, j*) in *V*(*G*) × *V*(*G*) such that *i, j, w* are all distinct. Then, the Betweenness Centrality is calculated as:

$$\boldsymbol{Cb}\left( \boldsymbol{w} \right)=\sum_{(\boldsymbol{i}, \boldsymbol{j})\in\boldsymbol{V}(\boldsymbol{w})} \frac{\boldsymbol{\sigma ij}(\boldsymbol{w})}{\boldsymbol{\sigma ij}}$$

**S5.5 Pagerank**

PageRank is a famous ranking algorithm that forms the basis of the Google™ search engine. In practice, PageRank assigns a score *s_i_* to denote the attractiveness of the webpage *i*. Webpage *i* obtains a higher score if many other important webpages point to it. From the physical perspective, PageRank describes a random walk process on a directed network, where the score *s_i_* is proportional to the frequency of visits to a particular node *i* by a random walker. In the PageRank algorithm, the parameter *c* (0 ≤ *c* ≤ 1) called *return probability* is introduced, which represents the probability for a random walker to jump to a random node and (1 − *c*) is the probability for the random walker to continue walking through the directed links. In this way, the node *i*'s centrality score at time *t* (*t* ≥ 1) is given by:

$$\boldsymbol{Si}\left( \boldsymbol{t} \right)=\boldsymbol{c}+\left( \mathbf{1}-\boldsymbol{c} \right)\sum_{\boldsymbol{j}=\mathbf{1}}^{\boldsymbol{N}} [\frac{\boldsymbol{Aij}}{\boldsymbol{K}_{\boldsymbol{j}}^{\boldsymbol{si}\left( \boldsymbol{t} \right)}}\left( \mathbf{1}-\boldsymbol{\delta}_{\boldsymbol{K}_{\boldsymbol{j}}^{\boldsymbol{si}\left( \boldsymbol{t} \right)}, \mathbf{0}} \right)+\frac{\mathbf{1}}{\boldsymbol{N}}\boldsymbol{\delta}_{\boldsymbol{K}_{\boldsymbol{j}}^{\boldsymbol{si}\left( \boldsymbol{t} \right)}, \mathbf{0}}]\boldsymbol{S}_{\boldsymbol{j}}(\boldsymbol{t}-\mathbf{1})$$

where δ*_a_*_,_ *_b_* = 1 when *a* = *b* and δ*_a_*_,_ *_b_* = 0 otherwise. Initially, we assign each node one random walker, namely *s_i_* (0) = 1 for *i*=1, 2, …, N. The typical value of the return probability in computer science is approximately 0.15. The final score of each node is defined as the steady value after the convergence of *s_i_*(*t*). The final ranking of nodes in PageRank, denoted as *R_p_*, will be obtained by sorting *s_i_* in a descending order when *s_i_* reaches the stable state ^10^.

**S5.5 Eigencentrality**

The heuristic behind the eigencentrality measure is based on the key idea that the centrality of a node is proportional to the sum of centralities of its neighbourhood and therefore, a node connected to central nodes, will also be central. As explained in the introductory section of this paper, this heuristic leads us to calculate the centrality of a node i by an equation of the form:

$$\boldsymbol{C}_{\boldsymbol{i}}=\frac{\mathbf{1}}{\boldsymbol{\lambda}_{\boldsymbol{max}}}\sum_{\boldsymbol{j\epsilon N}(\boldsymbol{i})} \boldsymbol{C}_{\boldsymbol{j}}=\frac{\mathbf{1}}{\boldsymbol{\lambda}_{\boldsymbol{max}}}\sum_{\boldsymbol{j}=\mathbf{1}}^{\boldsymbol{n}} \boldsymbol{A}_{\boldsymbol{ij}}\boldsymbol{c}_{\boldsymbol{j},} \boldsymbol{i}=\mathbf{1}, \mathbf{2}, \ldots, \boldsymbol{n}$$

where λmax is the largest eigenvalue of the adjacency matrix A. Note that the above equation leads directly to the eigenvector problem:

$\boldsymbol{A}_{\boldsymbol{c}}=\boldsymbol{\lambda}_{\boldsymbol{max}}\boldsymbol{c}$

where c exist and it is positive as consequence of the Perron-Frobenius theorem ^11^.

**S5.6 Maximal clique centrality (MCC)**

MCC was calculated by a Cytoscape plugin cytohubba and it has been proved to have a better performance on the precision to rank the importance of the nodes in the network than other topological analyses. Given a node v, the MCC of v is defined as MCC(v) = ∑C∈S(v)(|C| - 1)!, where S(v) is the collection of maximal cliques which contain v, and (|C|-1)! is the product of all positive integers less than |C| ^7^.

**Table S2. Number of nodes, edges, and** **frequency of** **multimorbidity patterns in the multimorbidity networks stratified by age and sex in China and the UK.**

| Age range | Multimorbidity networks | China | | | | | | UK | | | | | |
| --- | --- | --- | --- | --- | --- | --- | --- | --- | --- | --- | --- | --- | --- |
|  |  | Male | | | Female | | | Male | | | Female | | |
|  |  | Nodes (%) | Edges (%) | Fre* (%) | Nodes (%) | Edges (%) | Fre* (%) | Nodes (%) | Edges (%) | Fre* (%) | Nodes (%) | Edges (%) | Fre* (%) |
| 40  -  44 | Complete network | 279 | 735 | 31, 588 | 238 | 492 | 1, 4091 | 298 | 369 | 1, 597 | 183 | 197 | 2, 209 |
|  | Hub diseases’ network (Percentage^1^) | 10 (3.58) | 25 (3.40) | 5,684 (17.99) | 10 (4.20) | 18 (3.66) | 2,442 (17.33) | 10 (3.36) | 6 (1.63) | 177 (11.08) | 10 (5.46) | 9 (4.57) | 166 (7.51) |
|  | Hub diseases associated network (Percentage^2^) | 121 (43.37) | 238 (32.38) | 20,341 (64.39) | 97 (40.76) | 168 (34.15) | 9, 052 (64.24) | 57 (19.13) | 71 (19.24) | 739 (46.27) | 50 (27.32) | 61 (30.96) | 741 (33.54) |
| 45  -  49 | Complete network | 291 | 808 | 42, 716 | 277 | 646 | 23, 931 | 193 | 262 | 3, 563 | 170 | 222 | 4, 310 |
|  | Hub diseases’ network (Percentage^1^) | 10 (3.44) | 31 (3.84) | 11,130 (26.06) | 10 (3.61) | 19 (2.94) | 3,167 (13.23) | 10 (5.18) | 15 (5.73) | 738 (20.71) | 10 (5.88) | 13 (5.86) | 873 (20.26) |
|  | Hub diseases associated network (Percentage^2^) | 119 (40.89) | 248 (30.69) | 30,832 (72.18) | 109 (39.35) | 202 (31.27) | 14,526 (60.70) | 52 (26.94) | 82 (31.30) | 2,131 (59.81) | 49 (28.82) | 74 (33.33) | 2,697 (62.58) |
| 50  -  54 | Complete network | 298 | 773 | 43, 273 | 266 | 640 | 24, 408 | 200 | 311 | 6, 513 | 182 | 291 | 7, 408 |
|  | Hub diseases’ network (Percentage^1^) | 10 (3.36) | 27 (3.49) | 10,611 (24.52) | 10 (3.76) | 18 (2.81) | 4,089 (16.75) | 10 (5.00) | 22 (7.07) | 1,692 (25.98) | 10 (5.49) | 20 (6.87) | 1,347 (18.18) |
|  | Hub diseases’ associated network (Percentage^2^) | 118 (39.60) | 218 (28.20) | 31,576 (72.97) | 99 (37.22) | 193 (30.16) | 16,496 (67.58) | 58 (29.00) | 99 (31.83) | 4,047 (62.14) | 53 (29.12) | 95 (32.65) | 4,128 (55.72) |
| 55  -  59 | Complete network | 293 | 695 | 32, 102 | 256 | 552 | 19, 272 | 206 | 352 | 10, 036 | 200 | 347 | 9, 444 |
|  | Hub diseases network (Percentage^1^) | 10 (3.41) | 26 (3.74) | 6,416 (19.99) | 10 (3.91) | 20 (3.62) | 4,612 (23.93) | 10 (4.85) | 21 (5.97) | 2,072 (20.65) | 10 (5.00) | 13 (3.75) | 1,114 (11.80) |
|  | Hub diseases’ associated network (Percentage^2^) | 103 (35.15) | 194 (27.91) | 23,050 (71.80) | 83 (32.42) | 160 (28.99) | 14,532 (75.40) | 60 (29.13) | 106 (30.11) | 5,495 (54.75) | 62 (31.00) | 107 (30.84) | 5,507 (58.31) |

##1: The number of nodes/edges and frequency of multimorbidity patterns in the hub diseases network account for the complete network.

2: The number of nodes/edges and frequency of multimorbidity patterns in the hub diseases associated network accounts for the complete network.

Fre*: Frequency

**Table S3. The per-capita disease diagnoses of each chapter among each age from 40 to 59 years among Chinese male inpatients.**

| Disease chapters |  | Ages（years） | | | | | | | | | | | | | | | | | | | |
| --- | --- | --- | --- | --- | --- | --- | --- | --- | --- | --- | --- | --- | --- | --- | --- | --- | --- | --- | --- | --- | --- |
|  | 40-59 | 40 | 41 | 42 | 43 | 44 | 45 | 46 | 47 | 48 | 49 | 50 | 51 | 52 | 53 | 54 | 55 | 56 | 57 | 58 | 59 |
| Chapter1 | 0.06 | 0.07 | 0.08 | 0.07 | 0.07 | 0.07 | 0.07 | 0.06 | 0.06 | 0.06 | 0.06 | 0.05 | 0.06 | 0.05 | 0.06 | 0.06 | 0.05 | 0.04 | 0.05 | 0.05 | 0.05 |
| Chapter 2 | 0.05 | 0.04 | 0.05 | 0.05 | 0.04 | 0.05 | 0.05 | 0.05 | 0.05 | 0.06 | 0.05 | 0.05 | 0.06 | 0.06 | 0.06 | 0.05 | 0.07 | 0.07 | 0.07 | 0.07 | 0.06 |
| Chapter 3 | 0.03 | 0.03 | 0.03 | 0.03 | 0.03 | 0.03 | 0.03 | 0.03 | 0.02 | 0.03 | 0.02 | 0.03 | 0.03 | 0.03 | 0.02 | 0.03 | 0.03 | 0.03 | 0.03 | 0.03 | 0.02 |
| Chapter 4 | 0.31 | 0.27 | 0.26 | 0.26 | 0.26 | 0.28 | 0.27 | 0.28 | 0.31 | 0.32 | 0.31 | 0.32 | 0.34 | 0.34 | 0.35 | 0.36 | 0.39 | 0.37 | 0.37 | 0.38 | 0.38 |
| Chapter 5 | 0.02 | 0.02 | 0.02 | 0.02 | 0.02 | 0.02 | 0.02 | 0.02 | 0.02 | 0.02 | 0.01 | 0.02 | 0.02 | 0.02 | 0.01 | 0.01 | 0.01 | 0.01 | 0.01 | 0.01 | 0.01 |
| Chapter 6 | 0.12 | 0.10 | 0.11 | 0.10 | 0.10 | 0.11 | 0.11 | 0.11 | 0.11 | 0.12 | 0.12 | 0.12 | 0.13 | 0.13 | 0.13 | 0.13 | 0.12 | 0.13 | 0.13 | 0.12 | 0.13 |
| Chapter 7 | 0.06 | 0.05 | 0.05 | 0.05 | 0.05 | 0.05 | 0.05 | 0.05 | 0.05 | 0.06 | 0.06 | 0.06 | 0.06 | 0.06 | 0.06 | 0.07 | 0.07 | 0.07 | 0.07 | 0.07 | 0.06 |
| Chapter 8 | 0.04 | 0.04 | 0.04 | 0.04 | 0.04 | 0.05 | 0.05 | 0.04 | 0.04 | 0.04 | 0.04 | 0.04 | 0.04 | 0.04 | 0.04 | 0.04 | 0.04 | 0.04 | 0.05 | 0.05 | 0.04 |
| Chapter 9 | 0.72 | 0.45 | 0.46 | 0.50 | 0.52 | 0.55 | 0.57 | 0.62 | 0.66 | 0.68 | 0.72 | 0.82 | 0.81 | 0.86 | 0.88 | 0.99 | 1.02 | 0.99 | 1.07 | 1.09 | 1.10 |
| Chapter 10 | 0.21 | 0.21 | 0.21 | 0.22 | 0.22 | 0.20 | 0.20 | 0.21 | 0.21 | 0.21 | 0.22 | 0.19 | 0.21 | 0.21 | 0.21 | 0.21 | 0.23 | 0.22 | 0.22 | 0.24 | 0.23 |
| Chapter 11 | 0.44 | 0.47 | 0.48 | 0.45 | 0.44 | 0.44 | 0.45 | 0.47 | 0.45 | 0.44 | 0.43 | 0.43 | 0.42 | 0.41 | 0.41 | 0.39 | 0.42 | 0.42 | 0.41 | 0.40 | 0.40 |
| Chapter 12 | 0.02 | 0.03 | 0.03 | 0.03 | 0.02 | 0.02 | 0.03 | 0.03 | 0.03 | 0.03 | 0.03 | 0.02 | 0.02 | 0.02 | 0.02 | 0.02 | 0.02 | 0.02 | 0.02 | 0.02 | 0.02 |
| Chapter 13 | 0.19 | 0.16 | 0.18 | 0.18 | 0.18 | 0.18 | 0.19 | 0.19 | 0.19 | 0.20 | 0.19 | 0.20 | 0.20 | 0.20 | 0.21 | 0.21 | 0.21 | 0.20 | 0.21 | 0.19 | 0.20 |
| Chapter 14 | 0.16 | 0.17 | 0.15 | 0.17 | 0.16 | 0.16 | 0.15 | 0.16 | 0.16 | 0.16 | 0.16 | 0.15 | 0.16 | 0.16 | 0.17 | 0.17 | 0.17 | 0.17 | 0.16 | 0.16 | 0.18 |
| Total | 2.43 | 2.10 | 2.11 | 2.15 | 2.16 | 2.20 | 2.22 | 2.32 | 2.36 | 2.41 | 2.43 | 2.50 | 2.55 | 2.60 | 2.64 | 2.74 | 2.84 | 2.79 | 2.86 | 2.88 | 2.89 |

#The meaning of chapters can be seen in the supplementary materials S1

**Table S4. The per-capita disease diagnoses of each chapter among each age from 40 to 59 years among the UK male inpatients.**

| Disease chapters |  | Ages（years） | | | | | | | | | | | | | | | | | | | |
| --- | --- | --- | --- | --- | --- | --- | --- | --- | --- | --- | --- | --- | --- | --- | --- | --- | --- | --- | --- | --- | --- |
|  | 40-59 | 40 | 41 | 42 | 43 | 44 | 45 | 46 | 47 | 48 | 49 | 50 | 51 | 52 | 53 | 54 | 55 | 56 | 57 | 58 | 59 |
| Chapter1 | 0.006 | 0.011 | 0.005 | 0.008 | 0.007 | 0.005 | 0.006 | 0.007 | 0.006 | 0.007 | 0.006 | 0.007 | 0.004 | 0.007 | 0.005 | 0.007 | 0.007 | 0.005 | 0.004 | 0.004 | 0.005 |
| Chapter 2 | 0.05 | 0.03 | 0.03 | 0.03 | 0.03 | 0.03 | 0.04 | 0.04 | 0.04 | 0.04 | 0.05 | 0.05 | 0.05 | 0.05 | 0.05 | 0.05 | 0.05 | 0.06 | 0.06 | 0.06 | 0.06 |
| Chapter 3 | 0.01 | 0.01 | 0.01 | 0.01 | 0.01 | 0.01 | 0.01 | 0.01 | 0.01 | 0.01 | 0.01 | 0.01 | 0.02 | 0.01 | 0.01 | 0.01 | 0.01 | 0.01 | 0.01 | 0.01 | 0.01 |
| Chapter 4 | 0.06 | 0.03 | 0.04 | 0.04 | 0.04 | 0.05 | 0.05 | 0.05 | 0.05 | 0.05 | 0.06 | 0.06 | 0.06 | 0.07 | 0.07 | 0.06 | 0.07 | 0.07 | 0.08 | 0.08 | 0.08 |
| Chapter 5 | 0.03 | 0.03 | 0.03 | 0.04 | 0.04 | 0.04 | 0.04 | 0.04 | 0.04 | 0.04 | 0.03 | 0.04 | 0.04 | 0.03 | 0.03 | 0.03 | 0.03 | 0.03 | 0.03 | 0.03 | 0.03 |
| Chapter 6 | 0.04 | 0.05 | 0.05 | 0.04 | 0.05 | 0.05 | 0.04 | 0.05 | 0.05 | 0.04 | 0.05 | 0.05 | 0.04 | 0.04 | 0.04 | 0.04 | 0.04 | 0.04 | 0.04 | 0.04 | 0.04 |
| Chapter 7 | 0.05 | 0.03 | 0.03 | 0.03 | 0.04 | 0.05 | 0.04 | 0.04 | 0.04 | 0.04 | 0.04 | 0.04 | 0.05 | 0.05 | 0.05 | 0.05 | 0.06 | 0.05 | 0.06 | 0.06 | 0.06 |
| Chapter 8 | 0.01 | 0.01 | 0.02 | 0.02 | 0.02 | 0.01 | 0.02 | 0.02 | 0.01 | 0.01 | 0.02 | 0.02 | 0.01 | 0.02 | 0.02 | 0.01 | 0.02 | 0.02 | 0.02 | 0.01 | 0.01 |
| Chapter 9 | 0.29 | 0.16 | 0.14 | 0.15 | 0.19 | 0.18 | 0.19 | 0.22 | 0.23 | 0.23 | 0.26 | 0.26 | 0.28 | 0.29 | 0.30 | 0.31 | 0.32 | 0.34 | 0.35 | 0.37 | 0.38 |
| Chapter 10 | 0.10 | 0.14 | 0.12 | 0.12 | 0.12 | 0.11 | 0.11 | 0.11 | 0.11 | 0.11 | 0.12 | 0.10 | 0.10 | 0.11 | 0.10 | 0.09 | 0.09 | 0.10 | 0.10 | 0.09 | 0.09 |
| Chapter 11 | 0.40 | 0.36 | 0.37 | 0.37 | 0.37 | 0.39 | 0.41 | 0.41 | 0.40 | 0.40 | 0.40 | 0.40 | 0.41 | 0.40 | 0.40 | 0.41 | 0.44 | 0.41 | 0.39 | 0.40 | 0.39 |
| Chapter 12 | 0.07 | 0.10 | 0.09 | 0.09 | 0.08 | 0.07 | 0.08 | 0.07 | 0.08 | 0.07 | 0.07 | 0.07 | 0.07 | 0.07 | 0.07 | 0.06 | 0.06 | 0.07 | 0.06 | 0.06 | 0.06 |
| Chapter 13 | 0.22 | 0.24 | 0.25 | 0.22 | 0.23 | 0.23 | 0.23 | 0.23 | 0.23 | 0.23 | 0.23 | 0.22 | 0.22 | 0.21 | 0.23 | 0.21 | 0.21 | 0.20 | 0.21 | 0.20 | 0.21 |
| Chapter 14 | 0.12 | 0.11 | 0.12 | 0.12 | 0.11 | 0.12 | 0.12 | 0.11 | 0.10 | 0.12 | 0.11 | 0.11 | 0.11 | 0.12 | 0.11 | 0.12 | 0.12 | 0.13 | 0.13 | 0.13 | 0.14 |
| Total | 1.46 | 1.31 | 1.30 | 1.29 | 1.32 | 1.34 | 1.39 | 1.40 | 1.40 | 1.39 | 1.44 | 1.44 | 1.46 | 1.46 | 1.48 | 1.47 | 1.51 | 1.52 | 1.55 | 1.55 | 1.58 |

#The meaning of chapters can be seen in the supplementary materials S1

**Table S5. The per-capita disease diagnoses of each chapter among each age from 40 to 59 years among Chinese female inpatients.**

| Disease chapters |  | Ages（years） | | | | | | | | | | | | | | | | | | | |
| --- | --- | --- | --- | --- | --- | --- | --- | --- | --- | --- | --- | --- | --- | --- | --- | --- | --- | --- | --- | --- | --- |
|  | 40-59 | 40 | 41 | 42 | 43 | 44 | 45 | 46 | 47 | 48 | 49 | 50 | 51 | 52 | 53 | 54 | 55 | 56 | 57 | 58 | 59 |
| Chapter1 | 0.05 | 0.05 | 0.05 | 0.05 | 0.05 | 0.05 | 0.05 | 0.05 | 0.05 | 0.04 | 0.05 | 0.04 | 0.04 | 0.04 | 0.04 | 0.05 | 0.04 | 0.04 | 0.04 | 0.04 | 0.04 |
| Chapter 2 | 0.15 | 0.17 | 0.17 | 0.19 | 0.18 | 0.18 | 0.18 | 0.18 | 0.18 | 0.18 | 0.16 | 0.15 | 0.13 | 0.13 | 0.12 | 0.11 | 0.11 | 0.09 | 0.11 | 0.09 | 0.09 |
| Chapter 3 | 0.07 | 0.10 | 0.10 | 0.10 | 0.09 | 0.09 | 0.09 | 0.10 | 0.10 | 0.09 | 0.08 | 0.07 | 0.05 | 0.05 | 0.04 | 0.04 | 0.03 | 0.03 | 0.03 | 0.03 | 0.03 |
| Chapter 4 | 0.21 | 0.12 | 0.12 | 0.12 | 0.14 | 0.15 | 0.16 | 0.17 | 0.18 | 0.19 | 0.19 | 0.20 | 0.24 | 0.28 | 0.28 | 0.29 | 0.29 | 0.32 | 0.32 | 0.35 | 0.32 |
| Chapter 5 | 0.02 | 0.02 | 0.02 | 0.02 | 0.02 | 0.02 | 0.02 | 0.02 | 0.02 | 0.02 | 0.02 | 0.02 | 0.02 | 0.03 | 0.02 | 0.02 | 0.02 | 0.01 | 0.02 | 0.02 | 0.02 |
| Chapter 6 | 0.10 | 0.07 | 0.07 | 0.08 | 0.07 | 0.08 | 0.09 | 0.09 | 0.10 | 0.10 | 0.10 | 0.11 | 0.12 | 0.12 | 0.13 | 0.12 | 0.13 | 0.12 | 0.13 | 0.12 | 0.12 |
| Chapter 7 | 0.05 | 0.03 | 0.03 | 0.03 | 0.03 | 0.04 | 0.04 | 0.04 | 0.04 | 0.04 | 0.05 | 0.04 | 0.05 | 0.05 | 0.05 | 0.06 | 0.06 | 0.06 | 0.08 | 0.06 | 0.07 |
| Chapter 8 | 0.05 | 0.04 | 0.04 | 0.04 | 0.04 | 0.04 | 0.04 | 0.05 | 0.05 | 0.05 | 0.05 | 0.05 | 0.05 | 0.06 | 0.06 | 0.07 | 0.07 | 0.07 | 0.06 | 0.06 | 0.06 |
| Chapter 9 | 0.49 | 0.21 | 0.23 | 0.26 | 0.27 | 0.31 | 0.33 | 0.36 | 0.40 | 0.43 | 0.48 | 0.49 | 0.59 | 0.64 | 0.64 | 0.68 | 0.73 | 0.82 | 0.84 | 0.91 | 0.93 |
| Chapter 10 | 0.16 | 0.14 | 0.16 | 0.15 | 0.14 | 0.14 | 0.16 | 0.16 | 0.15 | 0.17 | 0.16 | 0.17 | 0.16 | 0.16 | 0.18 | 0.18 | 0.18 | 0.18 | 0.17 | 0.18 | 0.19 |
| Chapter 11 | 0.33 | 0.29 | 0.29 | 0.29 | 0.31 | 0.30 | 0.32 | 0.30 | 0.31 | 0.33 | 0.34 | 0.35 | 0.35 | 0.36 | 0.36 | 0.35 | 0.38 | 0.38 | 0.37 | 0.39 | 0.35 |
| Chapter 12 | 0.02 | 0.02 | 0.02 | 0.02 | 0.02 | 0.02 | 0.02 | 0.02 | 0.02 | 0.02 | 0.02 | 0.02 | 0.02 | 0.01 | 0.02 | 0.02 | 0.01 | 0.02 | 0.01 | 0.01 | 0.01 |
| Chapter 13 | 0.24 | 0.18 | 0.17 | 0.19 | 0.18 | 0.21 | 0.21 | 0.23 | 0.23 | 0.23 | 0.25 | 0.27 | 0.26 | 0.27 | 0.30 | 0.30 | 0.29 | 0.28 | 0.30 | 0.32 | 0.31 |
| Chapter 14 | 0.31 | 0.41 | 0.42 | 0.42 | 0.42 | 0.42 | 0.40 | 0.41 | 0.37 | 0.38 | 0.32 | 0.28 | 0.26 | 0.24 | 0.20 | 0.18 | 0.18 | 0.14 | 0.15 | 0.14 | 0.13 |
| Total | 2.25 | 1.86 | 1.88 | 1.95 | 1.97 | 2.05 | 2.09 | 2.15 | 2.19 | 2.26 | 2.27 | 2.25 | 2.34 | 2.43 | 2.44 | 2.46 | 2.50 | 2.56 | 2.63 | 2.70 | 2.69 |

#The meaning of chapters can be seen in the supplementary materials S1

**Table S6. The per-capita disease diagnoses of each chapter among each age from 40 to 59 years among the UK female inpatients.**

| Disease chapters |  | Ages（years） | | | | | | | | | | | | | | | | | | | |
| --- | --- | --- | --- | --- | --- | --- | --- | --- | --- | --- | --- | --- | --- | --- | --- | --- | --- | --- | --- | --- | --- |
|  | 40-59 | 40 | 41 | 42 | 43 | 44 | 45 | 46 | 47 | 48 | 49 | 50 | 51 | 52 | 53 | 54 | 55 | 56 | 57 | 58 | 59 |
| Chapter1 | 0.004 | 0.006 | 0.004 | 0.004 | 0.003 | 0.004 | 0.002 | 0.004 | 0.003 | 0.003 | 0.003 | 0.003 | 0.004 | 0.003 | 0.004 | 0.003 | 0.003 | 0.003 | 0.003 | 0.004 | 0.004 |
| Chapter 2 | 0.09 | 0.08 | 0.07 | 0.07 | 0.08 | 0.09 | 0.09 | 0.09 | 0.10 | 0.10 | 0.09 | 0.10 | 0.10 | 0.09 | 0.09 | 0.09 | 0.08 | 0.08 | 0.08 | 0.08 | 0.09 |
| Chapter 3 | 0.02 | 0.02 | 0.02 | 0.02 | 0.02 | 0.02 | 0.02 | 0.02 | 0.02 | 0.02 | 0.03 | 0.02 | 0.02 | 0.02 | 0.02 | 0.02 | 0.01 | 0.01 | 0.01 | 0.01 | 0.01 |
| Chapter 4 | 0.05 | 0.04 | 0.03 | 0.04 | 0.03 | 0.04 | 0.04 | 0.04 | 0.04 | 0.04 | 0.04 | 0.04 | 0.04 | 0.05 | 0.05 | 0.05 | 0.05 | 0.06 | 0.06 | 0.06 | 0.07 |
| Chapter 5 | 0.02 | 0.02 | 0.02 | 0.02 | 0.02 | 0.02 | 0.02 | 0.02 | 0.02 | 0.02 | 0.03 | 0.02 | 0.02 | 0.02 | 0.02 | 0.02 | 0.02 | 0.02 | 0.02 | 0.02 | 0.02 |
| Chapter 6 | 0.04 | 0.04 | 0.04 | 0.04 | 0.04 | 0.03 | 0.04 | 0.03 | 0.03 | 0.04 | 0.04 | 0.04 | 0.04 | 0.04 | 0.04 | 0.04 | 0.04 | 0.04 | 0.04 | 0.04 | 0.04 |
| Chapter 7 | 0.04 | 0.02 | 0.03 | 0.02 | 0.03 | 0.03 | 0.03 | 0.03 | 0.03 | 0.04 | 0.03 | 0.04 | 0.03 | 0.04 | 0.04 | 0.04 | 0.05 | 0.05 | 0.05 | 0.06 | 0.07 |
| Chapter 8 | 0.01 | 0.01 | 0.01 | 0.01 | 0.01 | 0.01 | 0.01 | 0.01 | 0.01 | 0.01 | 0.01 | 0.01 | 0.01 | 0.01 | 0.01 | 0.01 | 0.01 | 0.01 | 0.01 | 0.01 | 0.01 |
| Chapter 9 | 0.14 | 0.08 | 0.09 | 0.10 | 0.09 | 0.10 | 0.10 | 0.09 | 0.11 | 0.12 | 0.13 | 0.13 | 0.13 | 0.14 | 0.15 | 0.16 | 0.18 | 0.18 | 0.19 | 0.20 | 0.22 |
| Chapter 10 | 0.08 | 0.08 | 0.08 | 0.09 | 0.08 | 0.07 | 0.07 | 0.08 | 0.07 | 0.07 | 0.07 | 0.08 | 0.08 | 0.07 | 0.08 | 0.07 | 0.08 | 0.08 | 0.07 | 0.08 | 0.08 |
| Chapter 11 | 0.30 | 0.26 | 0.24 | 0.24 | 0.24 | 0.25 | 0.26 | 0.26 | 0.27 | 0.27 | 0.27 | 0.28 | 0.29 | 0.29 | 0.31 | 0.31 | 0.35 | 0.33 | 0.34 | 0.36 | 0.36 |
| Chapter 12 | 0.06 | 0.05 | 0.06 | 0.05 | 0.05 | 0.05 | 0.05 | 0.05 | 0.06 | 0.05 | 0.06 | 0.05 | 0.05 | 0.06 | 0.06 | 0.06 | 0.05 | 0.06 | 0.06 | 0.06 | 0.06 |
| Chapter 13 | 0.19 | 0.13 | 0.13 | 0.14 | 0.15 | 0.14 | 0.15 | 0.16 | 0.16 | 0.17 | 0.16 | 0.18 | 0.18 | 0.20 | 0.21 | 0.20 | 0.21 | 0.23 | 0.23 | 0.25 | 0.24 |
| Chapter 14 | 0.39 | 0.51 | 0.52 | 0.52 | 0.52 | 0.50 | 0.50 | 0.50 | 0.46 | 0.47 | 0.45 | 0.41 | 0.42 | 0.39 | 0.37 | 0.36 | 0.33 | 0.33 | 0.31 | 0.26 | 0.26 |
| Total | 1.43 | 1.33 | 1.34 | 1.37 | 1.37 | 1.36 | 1.37 | 1.39 | 1.37 | 1.41 | 1.40 | 1.41 | 1.42 | 1.42 | 1.44 | 1.43 | 1.46 | 1.46 | 1.48 | 1.49 | 1.53 |

#The meaning of chapters can be seen in the supplementary materials S1

**Table S7. The comparison of the percentage of inpatients with various disease conditions among Chinese and the UK inpatients by age and sex**

|  | 1 condition | 2 conditions | 3 conditions | 4 conditions | ≥5 conditions | Complex multimorbidity  (≥ 4 conditions) |
| --- | --- | --- | --- | --- | --- | --- |
| Chinese-male |  |  |  |  |  |  |
| 40-44 | 46.70 | 23.24 | 13.69 | 8.09 | 8.28 | 16.37 |
| 45-49 | 42.85 | 22.05 | 14.55 | 9.04 | 11.51 | 20.55 |
| 50-54 | 38.26 | 20.54 | 14.93 | 10.53 | 15.73 | 26.26 |
| 55-59 | 34.10 | 19.27 | 15.56 | 11.27 | 19.80 | 31.07 |
|  |  |  |  |  |  |  |
| UK-male |  |  |  |  |  |  |
| 40-44 | 76.28 | 18.15 | 4.16 | 1.02 | 0.39 | 1.41 |
| 45-49 | 71.81 | 19.84 | 5.80 | 1.72 | 0.83 | 2.55 |
| 50-54 | 68.92 | 20.97 | 6.84 | 2.19 | 1.08 | 3.27 |
| 55-59 | 65.02 | 22.51 | 8.12 | 2.92 | 1.44 | 4.36 |
|  |  |  |  |  |  |  |
| Chinese-female |  |  |  |  |  |  |
| 40-44 | 50.67 | 24.59 | 12.98 | 6.19 | 5.56 | 11.75 |
| 45-49 | 45.17 | 22.94 | 14.69 | 8.36 | 8.85 | 17.21 |
| 50-54 | 41.92 | 21.80 | 14.86 | 9.57 | 11.85 | 21.42 |
| 55-59 | 38.81 | 19.85 | 14.95 | 10.35 | 16.04 | 26.39 |
|  |  |  |  |  |  |  |
| UK- female |  |  |  |  |  |  |
| 40-44 | 73.84 | 19.44 | 5.10 | 1.10 | 0.51 | 1.61 |
| 45-49 | 72.07 | 20.20 | 5.50 | 1.59 | 0.63 | 2.22 |
| 50-54 | 70.33 | 20.77 | 6.22 | 1.86 | 0.82 | 2.68 |
| 55-59 | 67.55 | 21.92 | 7.09 | 2.30 | 1.14 | 3.44 |

**Table S8. The hub diseases in the male-specific multimorbidity network among China and the UK. (20 nodes)**

| ICD-10 | Diseases | Chapter | Degree  (China) | Degree  (UK) |
| --- | --- | --- | --- | --- |
| N40 | Hyperplasia of prostate | C14 | 38 |  |
| E87 | Other disorders of fluid, electrolyte and acid-base balance | C4 | 24 |  |
| E79 | Disorders of purine and pyrimidine metabolism | C4 | 20 |  |
| K76 | Other diseases of liver | C11 | 17 |  |
| E77 | Disorders of glycoprotein metabolism | C4 | 17 |  |
| J98 | Other respiratory disorders | C10 | 16 |  |
| N08 | Glomerular disorders in diseases classified elsewhere | C14 | 16 |  |
| A16 | Respiratory tuberculosis, not confirmed bacteriologically or histologically | C1 | 16 |  |
| J43 | Emphysema | C10 | 15 |  |
| I70 | Atherosclerosis | C9 | 14 |  |
| I50 | Heart failure | C9 |  | 18 |
| I10 | Essential (primary) hypertension | C9 |  | 13 |
| E78 | Disorders of lipoprotein metabolism and other lipidaemias | C4 |  | 13 |
| I21 | Acute myocardial infarction | C9 |  | 11 |
| I51 | Complications and ill-defined descriptions of heart disease | C9 |  | 10 |
| I48 | Atrial fibrillation and flutter | C9 |  | 9 |
| I44 | Atrioventricular and left bundle-branch block | C9 |  | 9 |
| F10 | Mental and behavioural disorders due to use of alcohol | C5 |  | 9 |
| I25 | Chronic ischaemic heart disease | C9 |  | 8 |
| J18 | Pneumonia, organism unspecified | C10 |  | 8 |

**Table S9. The hub diseases in the female-specific multimorbidity network among China and the UK. (14 nodes)**

| ICD-10 | Diseases | Chapter | Degree  (China) | Degree  (UK) |
| --- | --- | --- | --- | --- |
| D25 | Leiomyoma of uterus | C2 | 28 | 17 |
| N72 | Inflammatory disease of cervix uteri | C14 | 28 | 10 |
| N83 | Noninflammatory disorders of ovary, fallopian tube and broad ligament | C14 | 24 | 11 |
| N76 | Other inflammation of vagina and vulva | C14 | 23 |  |
| N70 | Salpingitis and oophoritis | C14 | 21 |  |
| N73 | Other female pelvic inflammatory diseases | C14 | 20 | 10 |
| N84 | Polyp of female genital tract | C14 | 17 | 11 |
| D64 | Other anaemias | C3 | 16 |  |
| K76 | Other diseases of liver | C11 | 15 |  |
| N85 | Other noninflammatory disorders of uterus, except cervix | C14 | 15 | 14 |
| N88 | Other noninflammatory disorders of cervix uteri | C14 |  | 15 |
| N80 | Endometriosis | C14 |  | 14 |
| J45 | Asthma | C10 |  | 10 |
| I10 | Essential (primary) hypertension | C9 |  | 9 |

**Table S10. The hub diseases in the 4 subpopulations of male inpatients among China and the UK. (26 nodes)**

| ICD-10 | Diseases | Chapter | 40-44Male | | 45-49Male | | 50-54Male | | 55-59Male | |
| --- | --- | --- | --- | --- | --- | --- | --- | --- | --- | --- |
|  |  |  | Degree  (China) | Degree  (UK) | Degree  (China) | Degree  (UK) | Degree  (China) | Degree  (UK) | Degree  (China) | Degree  (UK) |
| E78 | Dyslipidemia | C4 | 34 (R1)* | 7 (R8)* | 42 (R1)* | 9 (R5)* | 33 (R1)* | 14 (R2)* | 24 (R2)* | 12 (R4)* |
| I10 | Essential hypertension | C9 | 32 (R2)* | 7 (R5)* | 33 (R2)* | 16 (R1)* | 25 (R4)* | 20 (R1)* | 25 (R1)* | 19 (R1)* |
| K76 | Other diseases of liver | C11 | 31 (R3) |  | 31 (R3) |  | 29 (R2) |  | 23 (R5) |  |
| E87 | Other disorders of fluid, electrolyte and acid-base balance | C4 | 31 (R4) |  | 30 (R4) |  | 22 (R7) |  | 18 (R10) |  |
| I50 | Heart failure | C9 | 28 (R5) |  | 26 (R6)* | 10 (R4)* | 26 (R3)* | 14 (R3)* | 24 (R3)* | 13 (R3)* |
| E72 | Other disorders of amino-acid metabolism | C4 | 23 (R6) |  | 22 (R8) |  |  |  | 18 (R9) |  |
| K29 | Gastritis and duodenitis | C11 | 22 (R7)* | 9 (R1)* | 26 (R5)* | 10 (R3)* | 21 (R8)* | 11 (R5)* | 22 (R7)* | 11 (R8)* |
| I70 | Atherosclerosis | C9 | 21 (R8) |  | 26 (R7) |  | 25 (R5) |  | 24 (R4) |  |
| N18 | Chronic kidney disease | C14 | 21 (R9) |  |  |  |  |  |  |  |
| K74 | Fibrosis and cirrhosis of liver | C11 | 20 (R10) |  |  |  |  |  |  |  |
| N17 | Acute renal failure | C14 |  | 9 (R2) |  |  |  |  |  |  |
| J96 | Respiratory failure, not elsewhere classified | C10 |  | 9 (R3) |  |  |  |  |  |  |
| D70 | Agranulocytosis | C3 |  | 8 (R4) |  |  |  |  |  |  |
| K44 | Diaphragmatic hernia | C11 |  | 7 (R6) |  | 8 (R6) |  | 8 (R10) |  |  |
| I25 | Chronic ischaemic heart disease | C9 |  | 7 (R7) |  | 12 (R2) |  | 13 (R4) |  | 14 (R2) |
| F17 | Mental and behavioural disorders due to use of tobacco | C5 |  | 7 (R9) |  |  |  |  |  | 12 (R5) |
| H36 | Retinal disorders in diseases classified elsewhere | C7 |  | 7 (R10) |  |  |  |  |  |  |
| N40 | Hyperplasia of prostate | C14 |  |  | 22 (R9) |  | 25 (R6) |  | 23 (R6) |  |
| E11 | Type 2 diabetes mellitus | C4 |  |  | 21 (R10)* | 8 (R8)* | 20 (R9)* | 9 (R9)* |  |  |
| I21 | Acute myocardial infarction | C9 |  |  |  | 8 (R7) |  | 10 (R8) |  | 11 (R9) |
| F10 | Mental and behavioural disorders due to use of alcohol | C5 |  |  |  | 8 (R9) |  |  |  |  |
| I51 | Complications and ill-defined descriptions of heart disease | C9 |  |  |  | 8 (R10) |  | 11 (R7) |  | 12 (R7) |
| J98 | Other respiratory disorders | C10 |  |  |  |  | 19 (R10) |  |  |  |
| E66 | Obesity | C4 |  |  |  |  |  | 11 (R6) |  | 12 (R6) |
| J18 | Pneumonia, organism unspecified | C10 |  |  |  |  |  |  |  | 11 (R10) |
| I65 | Occlusion and stenosis of precerebral arteries, not resulting in cerebral infarction | C9 |  |  |  |  |  |  | 19 (R8) |  |

R+Number: this means the rank of the diseases according to degree in the corresponding complete multimorbidity networks.

*: this disease is overlapped hub diseases between China and the UK in the certain group of people.

**Table S11. The hub diseases in the 4 subpopulations of female inpatients among China and the UK. (28 nodes)**

| ICD-10 | Diseases | Chapter | 40-44Female | | 45-49Female | | 50-54Female | | 55-59Female | |
| --- | --- | --- | --- | --- | --- | --- | --- | --- | --- | --- |
|  |  |  | Degree  (China) | Degree  (UK) | Degree  (China) | Degree  (UK) | Degree  (China) | Degree  (UK) | Degree  (China) | Degree  (UK) |
| E78 | Dyslipidemia | C4 | 24 (R1)* | 6 (R9)* | 29 (R1)* | 8 (R8)* | 27 (R1)* | 10 (R9)* | 26 (R1)* | 11 (R5)* |
| K76 | Other diseases of liver | C11 | 23 (R2) |  | 22 (R5) |  | 27 (R2) |  | 21 (R2) |  |
| I50 | Heart failure | C9 | 21 (R3) |  | 26 (R3) |  | 25 (R3) |  | 19 (R4) |  |
| N72 | Inflammatory disease of cervix uteri | C14 | 19 (R4) |  | 23 (R4) |  | 20 (R6)* | 10 (R10)* | 16 (R8) |  |
| K29 | Gastritis and duodenitis | C11 | 18 (R5)* | 9 (R2)* | 21 (R6)* | 9 (R3)* | 20 (R5)* | 11 (R4)* | 16 (R5)* | 12 (R3)* |
| D25 | Leiomyoma of uterus | C2 | 17 (R6) |  |  | 9 (R2) | 17 (R8)* | 13 (R2)* | 16 (R7)* | 14 (R2)* |
| I10 | Essential hypertension | C9 | 17 (R7)* | 11 (R1)* | 26 (R2)* | 14 (R1)* | 22 (R4)* | 17 (R1)* | 19 (R3)* | 20 (R1)* |
| E87 | Other disorders of fluid, electrolyte and acid-base balance | C4 | 17 (R8) |  |  |  |  |  |  |  |
| N73 | Other female pelvic inflammatory diseases | C14 | 15 (R9)* | 6 (R6)* |  | 8 (R7) | 16 (R10) |  |  |  |
| D64 | Other anaemias | C3 | 15 (R10) |  | 18 (R8) |  | 17 (R9) |  |  |  |
| N80 | Endometriosis | C14 |  | 7 (R3) |  | 8 (R6) |  | 13 (R3) |  |  |
| E10 | Type 1 diabetes mellitus | C4 |  | 7 (R4) |  |  |  |  |  |  |
| K44 | Diaphragmatic hernia | C11 |  | 6 (R5) |  |  |  |  |  | 10 (R9) |
| E66 | Obesity | C4 |  | 6 (R7) |  |  |  |  |  | 11 (R6) |
| E11 | Type 2 diabetes mellitus | C4 |  | 6 (R8) |  |  |  |  |  |  |
| M47 | Spondylosis | C13 |  | 6 (R10) |  |  |  |  |  |  |
| I25 | Chronic ischaemic heart disease | C9 |  |  |  |  |  |  |  | 12 (R4) |
| N85 | Other noninflammatory disorders of uterus, except cervix | C14 |  |  |  | 9 (R4) |  | 11 (R5) |  |  |
| N83 | Noninflammatory disorders of ovary, fallopian tube and broad ligament | C14 |  |  |  | 8 (R5) |  | 10 (R8) |  |  |
| N92 | Excessive, frequent and irregular menstruation | C14 |  |  |  | 7 (R9) |  | 10 (R6) |  |  |
| N88 | Other noninflammatory disorders of cervix uteri | C14 |  |  |  | 7 (R10) |  |  |  |  |
| I63 | Cerebral infarction | C9 |  |  | 20 (R7) |  |  |  | 18 (R5) |  |
| N70 | Salpingitis and oophoritis | C14 |  |  | 18 (R9) |  |  |  |  |  |
| I70 | Atherosclerosis | C9 |  |  | 18 (R10) |  | 20 (R7) |  | 15 (R9) |  |
| N84 | Polyp of female genital tract | C14 |  |  |  |  |  | 10 (R7) |  | 10 (R8) |
| F17 | Mental and behavioural disorders due to use of tobacco | C5 |  |  |  |  |  |  |  | 11 (R7) |
| N81 | Female genital prolapse | C14 |  |  |  |  |  |  |  | 9 (R10) |
| I79 | Disorders of arteries, arterioles and capillaries in diseases classified elsewhere | C9 |  |  |  |  |  |  | 14 (R10) |  |

R+Number: this means the rank of the diseases according to degree in the corresponding complete multimorbidity networks.

*: this disease is overlapped hub diseases between China and the UK in the certain group of people.

**Figure S25. The total frequency of multimorbidity patterns associated with each ICD-10 chapter among sex-age-specific populations in China and the UK.**

**Figure S26. Property distribution for all nodes and nodes whose degree ranked top 10 among the male inpatients stratified by four age ranges in China and the UK.**

**Figure S27. Property distribution for all nodes and nodes whose degree ranked top 10 among the female inpatients stratified by four age ranges in China and the UK.**

**Supplementary summary**

We have uploaded the Supplementary_summary.xlsx to Google Drive, you can log in to the following link

(<https://docs.google.com/spreadsheets/d/1TE1Txasom_VQqRrP88WqNSdOmp8VWNq3/edit?usp=sharing&ouid=110854432052954855465&rtpof=true&sd=true>) to download the relevant specific information.

**References**

1. Organization WH. ICD-10 International Statistical Classification of Diseases and Related Health Problems. 05/03, 2023. Accessed 03 May, 2022. https://icd.who.int/browse10/2016/en#/

2. Yu J, Song F, Li Y, et al. Multimorbidity Analysis of 13 Systemic Diseases in Northeast China. International journal of environmental research and public health. Mar 11 2020;17(6)doi:10.3390/ijerph17061817

3. Zhang L, Li H, Su S, et al. Cohort Profile: The Shaanxi Blood Donor Cohort in China. Frontiers in cardiovascular medicine. 2022;9:841253. doi:10.3389/fcvm.2022.841253

4. Dodds RM, Bunn JG, Hillman SJ, et al. Simple approaches to characterising multiple long-term conditions (multimorbidity) and rates of emergency hospital admission: Findings from 495,465 UK Biobank participants. Journal of internal medicine. Jan 2023;293(1):100-109. doi:10.1111/joim.13567

5. Bao Y, Lu P, Wang M, et al. Exploring multimorbidity profiles in middle-aged inpatients: a network-based comparative study of China and the United Kingdom. BMC Med. Dec 13 2023;21(1):495. doi:10.1186/s12916-023-03204-y

6. Zhou X, Lin Q, Gui Y, Wang Z, Liu M, Lu H. Multimodal MR Images-Based Diagnosis of Early Adolescent Attention-Deficit/Hyperactivity Disorder Using Multiple Kernel Learning. Frontiers in neuroscience. 2021;15:710133. doi:10.3389/fnins.2021.710133

7. Chin CH, Chen SH, Wu HH, Ho CW, Ko MT, Lin CY. cytoHubba: identifying hub objects and sub-networks from complex interactome. BMC systems biology. 2014;8 Suppl 4(Suppl 4):S11. doi:10.1186/1752-0509-8-s4-s11

8. Pavlopoulos GA, Secrier M, Moschopoulos CN, et al. Using graph theory to analyze biological networks. BioData mining. Apr 28 2011;4:10. doi:10.1186/1756-0381-4-10

9. Costantini G, Perugini M. Generalization of clustering coefficients to signed correlation networks. PloS one. 2014;9(2):e88669. doi:10.1371/journal.pone.0088669

10. Yao L, Wei T, Zeng A, Fan Y, Di Z. Ranking scientific publications: the effect of nonlinearity. Scientific reports. 2014/10/17 2014;4(1):6663. doi:10.1038/srep06663

11. Alvarez-Socorro AJ, Herrera-Almarza GC, González-Díaz LA. Eigencentrality based on dissimilarity measures reveals central nodes in complex networks. Scientific reports. Nov 25 2015;5:17095. doi:10.1038/srep17095
